# Tumor exosomal circPTBP3 drives gastric cancer peritoneal metastasis via mesothelial-mesenchymal transition

**DOI:** 10.1038/s41419-025-07749-z

**Published:** 2025-06-11

**Authors:** Chao Dong, Yajing Zhou, Xiaochun Shen, Shuo Hu, Kaipeng Duan, Tao Chen, Weikang Li, Xiaotong Sun, Peiyuan Li, Pengbo Wang, Ye Han, Dongbao Li, Qiaoming Zhi, Jin Zhou

**Affiliations:** 1 Department of General Surgery, The First Affiliated Hospital of Soochow University, 188 Shizi Street, Suzhou, 215006 Jiangsu Province China; 2 Department of Pulmonary and Critical Care Medicine, The First Affiliated Hospital of Soochow University, 188 Shizi Street, Suzhou, 215006 Jiangsu Province China

**Keywords:** Metastasis, Tumour biomarkers, Non-coding RNAs, Cancer microenvironment, Gastric cancer

## Abstract

The peritoneum is the most common site of metastasis in advanced gastric cancer (GC), and the mechanisms underlying this process of gastric cancer peritoneal metastasis (GCPM) remain largely elusive. Mesothelial-mesenchymal transition (MMT) plays a crucial role in the progression of GCPM. In our current study, the data confirmed that GC-derived exosomes could significantly promote peritoneal metastasis through an MMT-dependent manner in vivo and in vitro. Using RNA-seq, we successfully identified a key circular RNA (circPTBP3). The expression of exosomal circPTBP3 in the plasma of GCPM patients was significantly upregulated and closely correlated with tumor differentiation, depth of invasion, lymphatic invasion, peritoneal metastasis, and TNM stage. Exosomal circPTBP3 thus serves as a reliable diagnostic and prognostic indicator in GCPM patients. Mechanistically, exosomal circPTBP3 could effectively promote the MMT phenotype of mesothelial cells in vitro. Located in the nucleus, circPTPB3 was found to recruit transcription factor AP-2-beta (TFAP2B) to the serum- and glucocorticoid-inducible kinase 1 (SGK1) promoter sites, thereby initiating its transcription in mesothelial cells. These findings suggest that exosomal circPTPB3 functions as a pivotal mediator in facilitating the interplay between GC cells and mesothelial cells, and it provides a promising diagnostic indicator and therapeutic target for GCPM patients.

## Introduction

The peritoneum is the most common site of metastasis in advanced gastric cancer (GC). Gastric cancer peritoneal metastasis (GCPM) occurs in nearly one-third of GC patients at diagnosis and accounts for more than half of distant metastases [1, 2]. Although significant progress has been made in the treatment of GCPM over the past two decades, including palliative systemic chemotherapy, cytoreductive surgery (CRS), and a wide variety of intraperitoneal chemotherapy, the prognosis for patients with GCPM remains dismal, with a median overall survival (OS) of less than one year [3, 4]. Meanwhile, conventional imaging techniques, such as endoscopic ultrasonography (EUS), computed tomography (CT), magnetic resonance imaging (MRI), and 18F-fluorodeoxyglucose positron emission tomography (18F-FDG PET), have limitations, and cannot accurately detect or measure GCPM, especially for diagnosing occult peritoneal metastasis [5]. The concealment and complexity of GCPM pose significant challenges to clinical diagnosis and treatment. Therefore, it is urgent to explore the molecular mechanism underlying GCPM and establish reliable diagnostic indicators or therapeutic targets.

Peritoneal metastasis (PM) is a complex process due to the heterogeneity of tumors and the complexity of the tumor microenvironment. Recent data indicate that molecular steps from primary tumor sites to PM include: (1) shedding of cancer cells in situ due to changes in epithelial junction and cytoskeleton; (2) adaptation of exfoliated cancer cells to the ascites microenvironment through resistance to anoikis and immune surveillance; (3) adhesion of cancer cells to mesothelial cells or entry into “milky spots”; and (4) invasion of tumor cells into the stroma and formation of micrometastases [6, 7]. The peritoneum is composed of a thin layer of mesothelial cells (MCs) lining a connective tissue that includes adipocytes, immune cells, fibroblasts, and capillaries [8]. Traditionally, peritoneal mesothelial cells (PMCs) which have epithelial characteristics have been considered an important barrier to intraperitoneal implantation and metastasis of tumors [9]. However, recent evidence suggests that PMCs can be transformed into carcinoma-associated fibroblasts (CAFs) through mesothelial-mesenchymal transition (MMT), and actively promote tumor progression. This MMT can also be considered a peritoneum-specific process of epithelial-mesenchymal transition (EMT) during peritoneal carcinomatosis [10, 11]. In GC, PMCs undergoing MMT can provide a favorable environment for metastatic tumor cells [12]. However, these studies are limited, and the mechanisms underlying MMT in GCPM remain largely unknown.

Circular RNAs (circRNAs) are a type of non-coding RNAs with a single-stranded covalently closed loop, that do not encode proteins but are known to regulate tumor initiation and progression, including hepatocellular cancer [13], colorectal cancer [14, 15], and prostate cancer [16]. In GC, emerging circRNA candidates have also been shown to play crucial roles in various biological processes, impacting tumorigenesis, progression, diagnosis, and therapy resistance [17–19]. Additionally, these circRNAs can be transferred by exosomes, which facilitate communications between GC cells and tumor microenvironment [20]. For instance, circSTAU2 can be packaged in exosomes and delivered to recipient cells, acting as a sponge for miR-589 to suppress the GC progression [21]. Plasma exosomal hsa_circ_0079439 has been found to be upregulated in GC patients and functions as a potential biomarker for both the early and late-stage GC diagnosis [22]. Similarly, differentially expressed exosomal circRNAs, such as circKIAA1797, circGMPS, circATP8A1, and circSTRBP, have been identified [23–26]. However, the regulation of circRNAs in GCPM and their specific mechanisms in MMT remain poorly understood.

In our current study, we firstly demonstrated that GC-derived exosomes significantly promote GCPM through an MMT-dependent manner both in vivo and in vitro. To further explore the potential transported materials, RNA-seq was performed, and circPTPB3 was identified for further investigation. Next, we systematically evaluated the clinical significance of exosomal circPTBP3 in GCPM patients and investigated its effects on MMT in vitro. Mechanistically, we provided reliable evidence that circPTBP3 enhances the transcription of serum- and glucocorticoid-inducible kinase 1 (SGK1) by recruiting transcription factor AP-2-beta (TFAP2B) in mesothelial cells. Finally, we confirmed that exosomal circPTBP3 promotes GCPM by inducing peritoneal fibrosis in vivo. Our data suggest that exosomal circPTPB3 serves as a key mediator in facilitating interrelationships between GC cells and PMCs and is a promising biomarker for diagnosing GCPM.

## Results

### GC cell-derived exosomes promoted GCPM in vivo

**Fig. 1:**
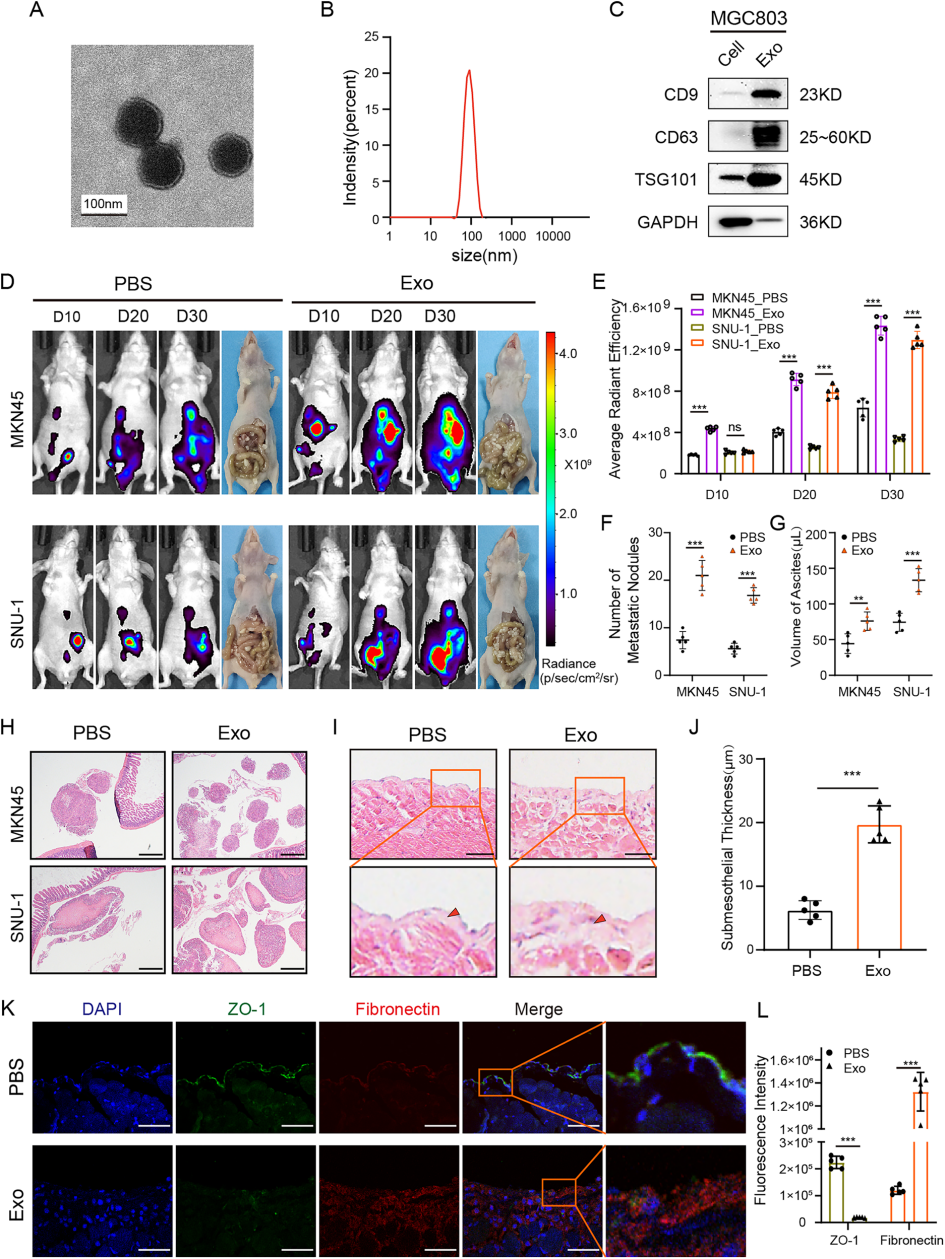
GC cell-derived exosomes promoted GCPM in vivo. **A** Exosome imaging by transmission electron microscopy. Scale bars, 100 μm. **B** Exosome particle size analysis. **C** Identification of exosome markers by western blot. Exo, MGC803 exosomes; cell, MGC803 cell lysates. **D, E** IVIS imaging of mice intraperitoneally injected with MKN45-luc or SNU-1-luc tumor cells on day10, 20 and 30, and corresponding statistical analyses. **F, G** Statistics of tumor nodules and ascites in the abdominal cavity of mice. **H** Microscopic metastatic nodules in the abdominal cavity. **I, H** The peritoneum of mice stained with HE and subcutaneous collagen thickness statistics. **K, L** Immunofluorescence staining and fluorescence intensity statistics of the peritoneal mesenchymal layer and collagen layer. Green, ZO-1; red, fibronectin. Scale bars, 50 μm. (*p < 0.05; **p < 0.01; ***p < 0.001; ns, no significance).

First, exosomes derived from MGC803 cells were successfully extracted and purified using differential ultracentrifugation. The characteristics of GC cell-derived exosomes, including their typical lipid bilayer structure, size range (50–141 nm), and enriched markers (CD9, CD63, and TSG101), were observed and identified using transmission electron microscope, nanoparticle tracking analysis, and western blot, respectively (Fig. 1A–C). After preconditioning the abdominal cavity of mice with exosomes or PBS for 7 days, we intraperitoneally injected MKN45 and SNU-1 GC cells to establish an intraperitoneal metastasis model. Fluorescence monitoring was performed using IVIS on days 10, 20, and 30. Fluorescence imaging showed that exosomes derived from MGC803 cells significantly enhanced the formation of abdominal metastases compared to the PBS control group (Fig. 1D, E). The subsequent dissection of metastatic nodes and collection of ascites both yielded positive results (Fig. 1F, G). Microscopically, more metastatic nodules were observed in the exosomes-treated group than in the PBS control group in the stained sections of peritoneal metastatic tissues (Fig. 1H).

To further explore how GC cell-derived exosomes influence the metastatic microenvironment, we intraperitoneally injected nude mice with exosomes or PBS and sacrificed them 7 days later. Hematoxylin and eosin (HE) staining was performed on sections of peritoneal specimens to observe the changes in peritoneal structure. Compared to the PBS group, the peritoneum of mice in the exosomes-treatment group was significantly disrupted and the subcutaneous collagen fiber layer was markedly thickened (Fig. 1I, J). Immunofluorescence staining for the epithelial phenotype maker (ZO-1) and mesenchymal phenotype marker (fibronectin) showed that exosomes induced a loss of epithelial phenotype, and a corresponding increase in mesenchymal phenotype in mice peritoneum (Fig. 1K, L). Further investigation revealed that GC exosomes increased the infiltration of mesothelial cell-derived cancer-associated fibroblasts (CAFs) into the peritoneum (Fig. S1). These data initially implied that MGC803-derived exosomes could promote GCPM by inducing peritoneal fibrosis in vivo.

### GC cell-derived exosomes promoted the mesenchymal transition of mesothelial cells in vitro

**Fig. 2:**
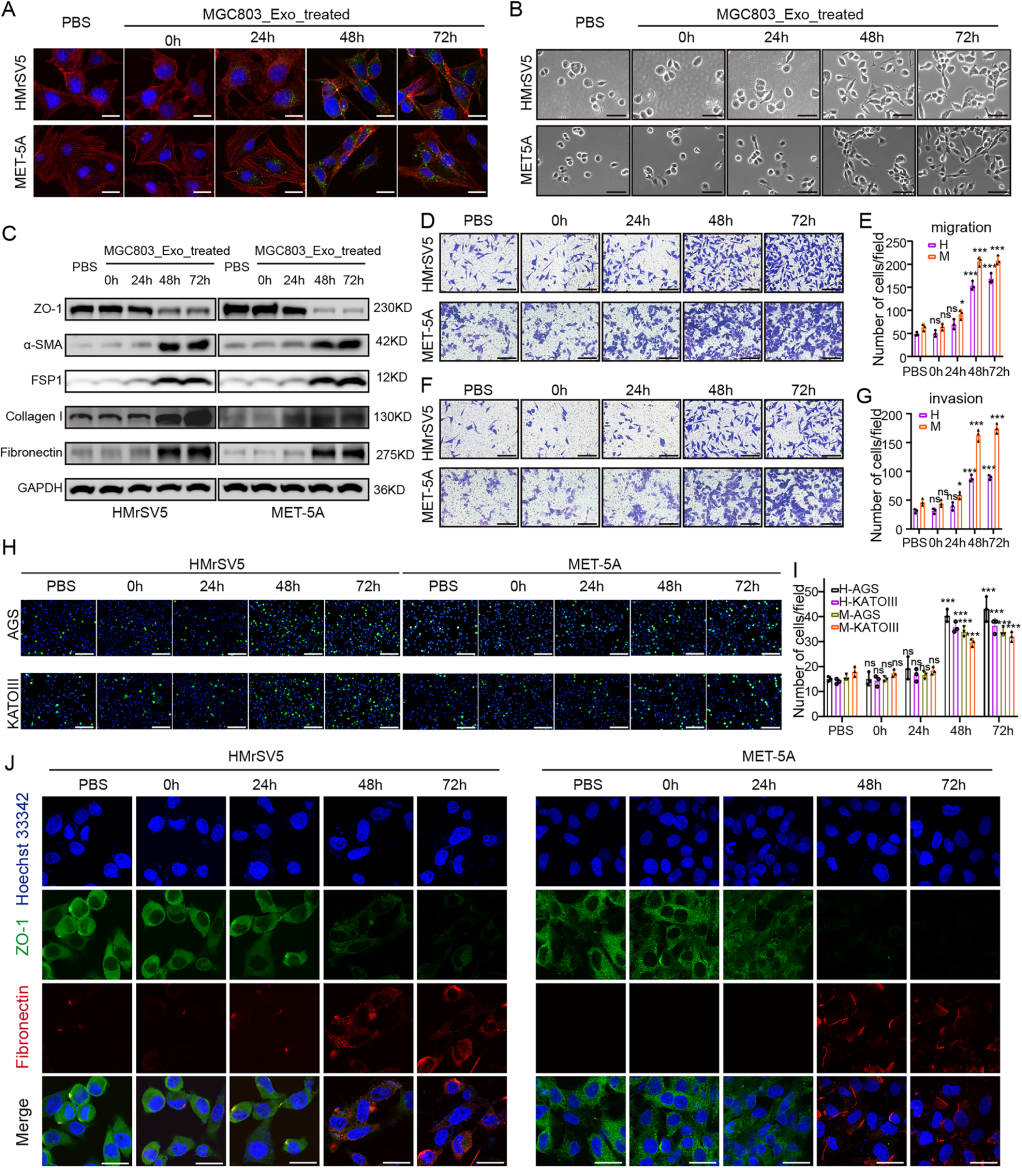
GC cell-derived exosomes promoted the mesenchymal transition of mesothelial cells in vitro. **A** Mesothelial cells were co-cultured with exosomes after 0, 24, 48, and 72 h. Blue, Hoechst33342, nucleus of mesothelial cells; green, PKH67, MGC803 exosomes; red, phalloidin. Scale bars, 20 μm. **B** Microscopic morphology of mesothelial cells. Bar, 40 μm. **C** Detection of epithelial and mesenchymal proteins (ZO-1, FSP1, α-SMA, Collagen I, and Fibronectin) in mesothelial cells co-cultured with exosomes by western blot analysis. **D, E** Observation and statistics of mesothelial cells migrating into the bottom surface of the chamber. Scale bars, 100 μm. **F, G** Observation and statistics of mesothelial cells invading the bottom surface of the chamber. Scale bars, 100 μm. **H, I** Observation and statistics of GC cells adhering to the mesothelial cell surface. Scale bars, 200 μm. **J** Immunofluorescence imaging of epithelial and mesenchymal markers of mesothelial cells. Blue, Hoechst33342, nucleus of mesothelial cells; green, ZO-1; red, Fibronectin. Scale bars, 10 μm. (*p < 0.05; **p < 0.01; ***p < 0.001; ns, no significance).

To verify the potential effects of GC cell-derived exosomes on mesothelial cells, HMrSV5 and MET-5A cells were co-cultured with exosomes labeled with the green fluorescent dye PKH67. Fluorescence imaging showed that the rapid uptake of exosomes by mesothelial cells occurred mostly after 48 or 72 h (Fig. 2A). Interestingly, the morphology of mesothelial cells shifted from a cobblestone appearance to a spindle shape after 48–72 h of co-culture (Fig. 2B). Western blot analysis revealed that the expression of epithelial marker protein ZO-1 was downregulated, while the expression of mesenchymal-related proteins, such asα-SMA, FSP1, Collagen I and Fibronectin, was significantly upregulated after 48 h of co-culture with exosomes (Fig. 2C). Similarly, subsequent in vitro experiments demonstrated that GC cell-derived exosomes significantly enhanced the migratory, invasive and adhesive (adhesion to AGS or KATOIII cells) abilities of mesothelial cells (Fig. 2D–I). Additionally, immunofluorescence assay confirmed the changes of ZO-1 and Fibronectin in the two mesothelial cell lines after co-culture with extracted exosomes (Fig. 2J). More representatively, GC exosomes significantly enhanced the expression of CAF markers in mesothelial cells (Fig S2). These results strongly demonstrate that MGC803-derived exosomes promote the mesothelial-mesenchymal transition (MMT) of mesothelial cells in vitro.

### Identification and clinical significance of exosomal circPTBP3 in GCPM patients

**Fig. 3:**
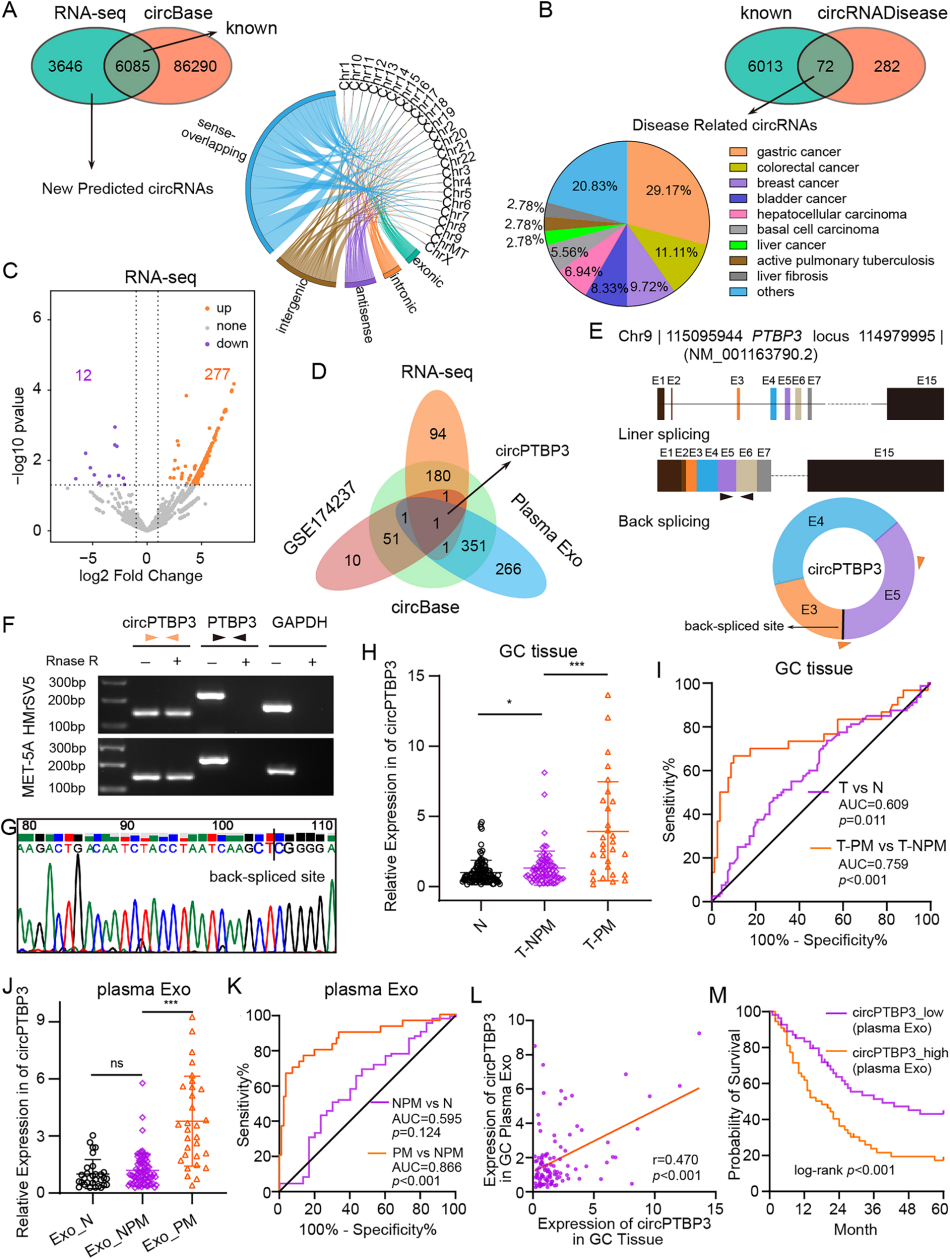
Identification and clinical significance of exosomal circPTBP3 in GCPM patients. **A** New prediction circRNA classification and origin tracing by RNA-seq. **B** Roles of known circRNAs from RNA-seq in disease. **C** Volcano plot of differentially expressed circRNAs in RNA-seq. **D** Volcano plot of differentially expressed circRNAs in RNA-seq. **E** Venn analysis of up-regulated circRNAs in the three datasets (RNA-seq, GSE174237, plasma exosomal circRNA data of GC patients). **E** Schematic representation of circPTBP3 origin and back-splicing. Black arrow, primers for linear product; orange arrow, primers for circPTBP3. **F** Assay of circRNA resistance to Rnase R. **G** Identification of the backsplice site of circPTBP3 by sanger sequencing. **H** Detection of circPTBP3 expression in GC and adjacent normal tissues. N, adjacent normal tissues; T-NPM, GC tissues in situ without peritoneal metastasis; T-PM, GC in situ with peritoneal metastasis. **I** ROC curves showed the efficacy of circPTBP3 expression on GC or GCPM. AUC, area under curve. **J** Detection of circPTBP3 expression in plasma exosomes from GC patients and healthy volunteers. Exo_N, exosomes from healthy volunteers; Exo_NPM, exosomes from GC patients without peritoneal metastasis; Exo_PM, exosomes from GC patients with peritoneal metastasis. **K** ROC curves detecting the efficacy of plasma exosomal circPTBP3 expression on GC or GCPM. (**L** Correlation analysis of the expression levels of circPTBP3 between tissue and plasma exosomes of GC patients. **M** The Kaplan-Meier method was employed to evaluate the prognosis of plasma exosomal circPTBP3 expression in GC patients. (*p < 0.05; **p < 0.01; ***p < 0.001; ns, no significance).

Since tumor-derived exosomes have been shown to promote GCPM in vivo and MMT in vitro, these results strongly motivate us to explore the involved molecules and possible mechanisms in GCPM. To this end, we extracted RNA from HMrsV5 cells after co-incubation with exosomes and performed RNA-seq. The analysis identified 9731 circRNAs with a median length of 640.5 nucleotides (Fig. S3A). Of these, 6085 circRNAs were known while the remaining 3646 were newly predicted and primarily derived from gene-coding strand localized on different chromosomes (Fig. 3A). By integrating the 6085 known circRNAs with the circDisease database, we identified 72 circRNAs that might be closely correlated with various diseases, particularly digestive system tumors (Fig. 3B). By analyzing the circRNA expression profile, we identified a total of 289 differentially expressed circRNAs, including 277 upregulated and 12 downregulated circRNAs. These findings were displayed by heatmap and volcano plot, respectively and the 12 top differentially expressed circRNAs were further verified by our subsequent RT-PCR analysis (Fig. S3B-C and Fig. 3C). Additionally, we incorporated available sequencing data from GC tissues (Fig. S3D-E) and plasma exosome into Venn analysis, ultimately identifying our most interesting molecule, circPTBP3 (Fig. 3D).

CircPTBP3 is derived from the NM_001163793.2 transcript of PTBP3 locus on chromosome 9 and is primarily formed through back-splicing of exons 3, 4 and 5 (Fig. 3E). Coincidentally, Alu elements flanking the intron of circPTBP3 within the PTBP3 gene are capable of reverse complementation, which may promote the splicing of circPTBP3 (Fig. S3F). RNA extracted from HMrSV5 and MET5A cells was treated with RNase R, and gel electrophoresis showed that circPTBP3 could be amplified and was resistant to RNase R digestion. Meanwhile, the presence of the back-splice junction of circPTBP3 was confirmed by Sanger sequencing (Fig. 3F, G). These data suggest that circPTBP3 is derived from its host gene PTBP3, and the stable loop structure based on head-to-tail splicing is the key element of circPTBP3.

Next, we evaluated the clinical significance of circPTBP3 derived from tumor tissues and plasma exosomes in both GC and GCPM patients. PCR analysis revealed that the circPTBP3 expression in GC tissues was significantly higher than that in adjacent normal tissues. More importantly, circPTBP3 levels in GCPM tissues were further elevated, compared to those in GC tissues (Fig. 3H). Unfortunately, the subsequent ROC curve showed relatively unsatisfactory results for the diagnostic potential of tissue circPTBP3 expression in GC and GCPM (AUC = 0.759, *p* < 0.001) (Fig. 3I). Furthermore, we detected circPTBP3 expression in plasma exosomes from 30 healthy volunteers and 110 involved GC patients. The results showed that circPTBP3 in plasma exosomes from GCPM patients was significantly higher than those in the other two groups (Fig. 3J). Interestingly, plasma exosomal circPTBP3 expression demonstrated a positive and satisfactory diagnostic role for GCPM patients (AUC = 0.866, *p* < 0.001) (Fig. 3K). Spearman correlation analysis also strongly indicated that circPTBP3 expression in GC plasma exosomes was highly positively correlated with corresponding GC tissue expression (*r* = 0.470, *p* < 0.001) (Fig. 3L).

Based on GC plasma exosomal circPTBP3 expressions, we divided the patients into subgroups and analyzed the relationships between exosomal circPTBP3 levels and GC-related clinicopathological features. Our data suggested that elevated exosomal circPTBP3 expression was significantly associated with tumor differentiation, depth of invasion, lymphatic invasion, peritoneal metastasis, and TNM stage (all *p* < 0.05) (Table S1). Additionally, Kaplan-Meier survival analysis indicated that a high plasma exosomal circPTBP3 level was associated with a relatively shorter overall survival (OS) rate (*p* < 0.001) (Fig. 3M). Univariate analysis for OS showed that tumor differentiation, depth of invasion, peritoneal metastasis, TNM stage, and high plasma exosomal circPTBP3 expression were all prognostic factors for poor prognosis (all *p* < 0.05). Multivariate analysis further revealed that high plasma exosomal circPTBP3 expression retained as an independent and significant prognostic factor for survival (relative risk, 1.763; 95% CI, 1.042–2.092; *p* = 0.035) (Table S2).

### CircPTBP3 was critical for exosomes-mediated mesenchymal transition of mesothelial cells

**Fig. 4:**
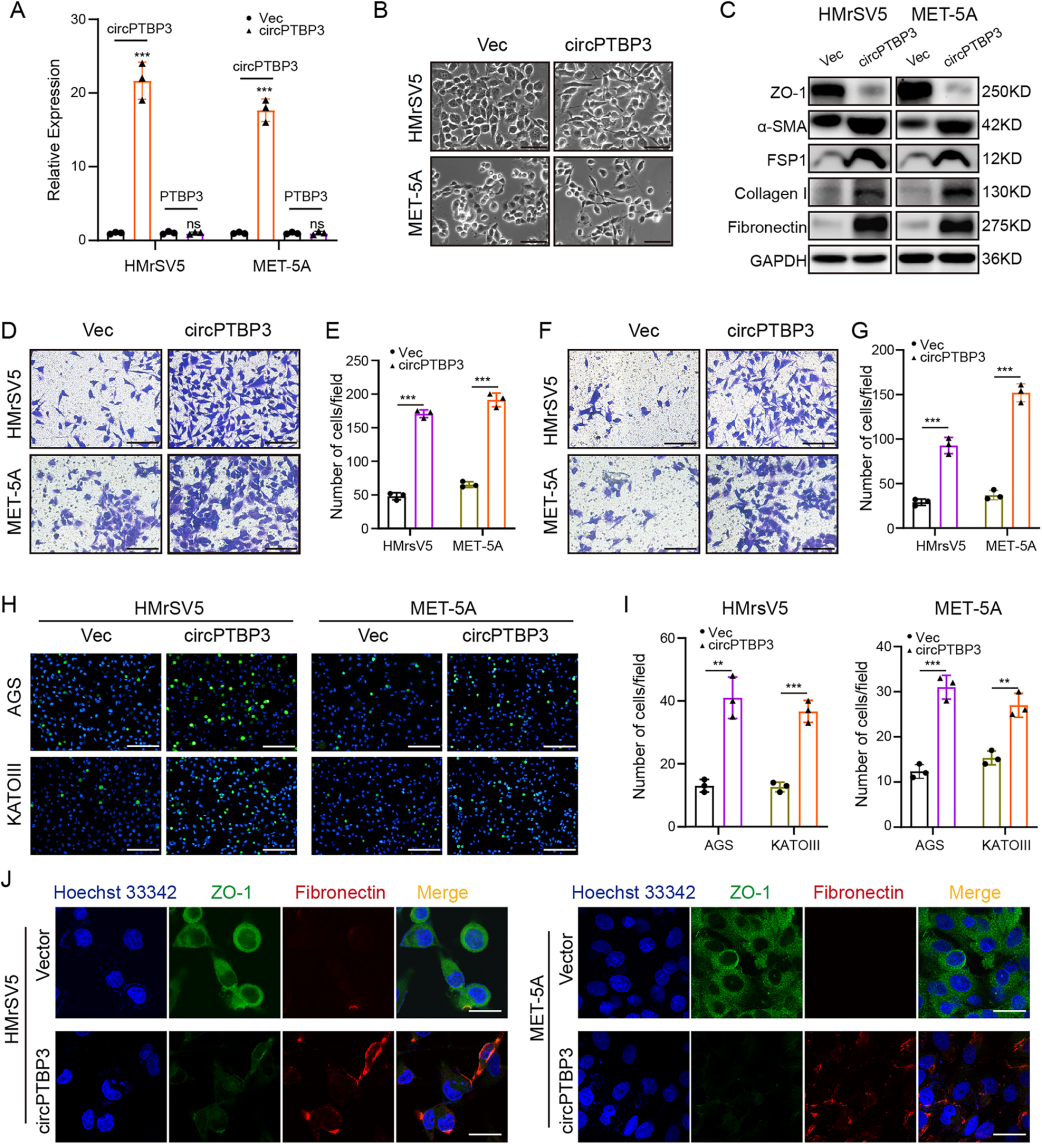
circPTBP3 overexpression induced the MMT of mesothelial cells in vitro. **A** The circPTBP3 overexpression vector was constructed and transfected into mesothelial cells. The PCR analysis was performed to detect the expressing changes of circPTBP3 and its host gene PTPB3 in HMrSV5 and MET-5A cells. Vec, control vector group; circPTBP3, circPTBP3 plasmid group. **B** Microscopic morphology of mesothelial cells. Bar, 40 μm. **C** Detection of epithelial and mesenchymal proteins (ZO-1, FSP1, α-SMA, Collagen I, and Fibronectin) in mesothelial cells by western blot. **D, E** Observation and statistics of mesothelial cells migrating into the bottom surface of the chamber. Scale bars, 100 μm. **F, G** Observation and statistics of mesothelial cells invading the bottom surface of the chamber. Scale bars, 100 μm. **H, I** Observation and statistics of GC cells adhering to the mesothelial cell surface. Scale bars, 200 μm. **J** Immunofluorescence imaging of epithelial and mesenchymal markers of mesothelial cells. Blue, Hoechst33342, nucleus of mesothelial cells; green, ZO-1; red, fibronectin. Scale bars, 10 μm. (*p < 0.05; **p < 0.01; ***p < 0.001; ns, no significance).

To elucidate the function of circPTBP3 in mesothelial cells, we successfully constructed a circPTBP3 overexpression vector. PCR results indicated that this vector efficiently expressed circPTBP3, thus being suitable for subsequent studies. Importantly, overexpression of circPTBP3 did not affect the expression of its host gene PTBP3 in either HMrSV5 or MET-5A cells (Fig. 4A). Similar to the results of GC cell-derived exosome treatment, the morphology of HMrSV5 and MET-5A cells shifted from a cobblestone appearance to a spindle shape following circPTBP3 overexpression (Fig. 4B). Subsequent experiments demonstrated that circPTBP3 significantly altered the expressions of mesothelial-mesenchymal transition (MMT)-related proteins (ZO-1, α-SMA, FSP1, Collagen I and Fibronectin) and enhanced the migratory, invasive and adhesive (adhesion to AGS or KATOIII cells) capabilities of mesothelial cells in vitro (Fig. 4C–J and Fig. S4). These findings preliminarily suggest that circPTBP3 may act as a potential inducer of MMT in mesothelial cells.

**Fig. 5:**
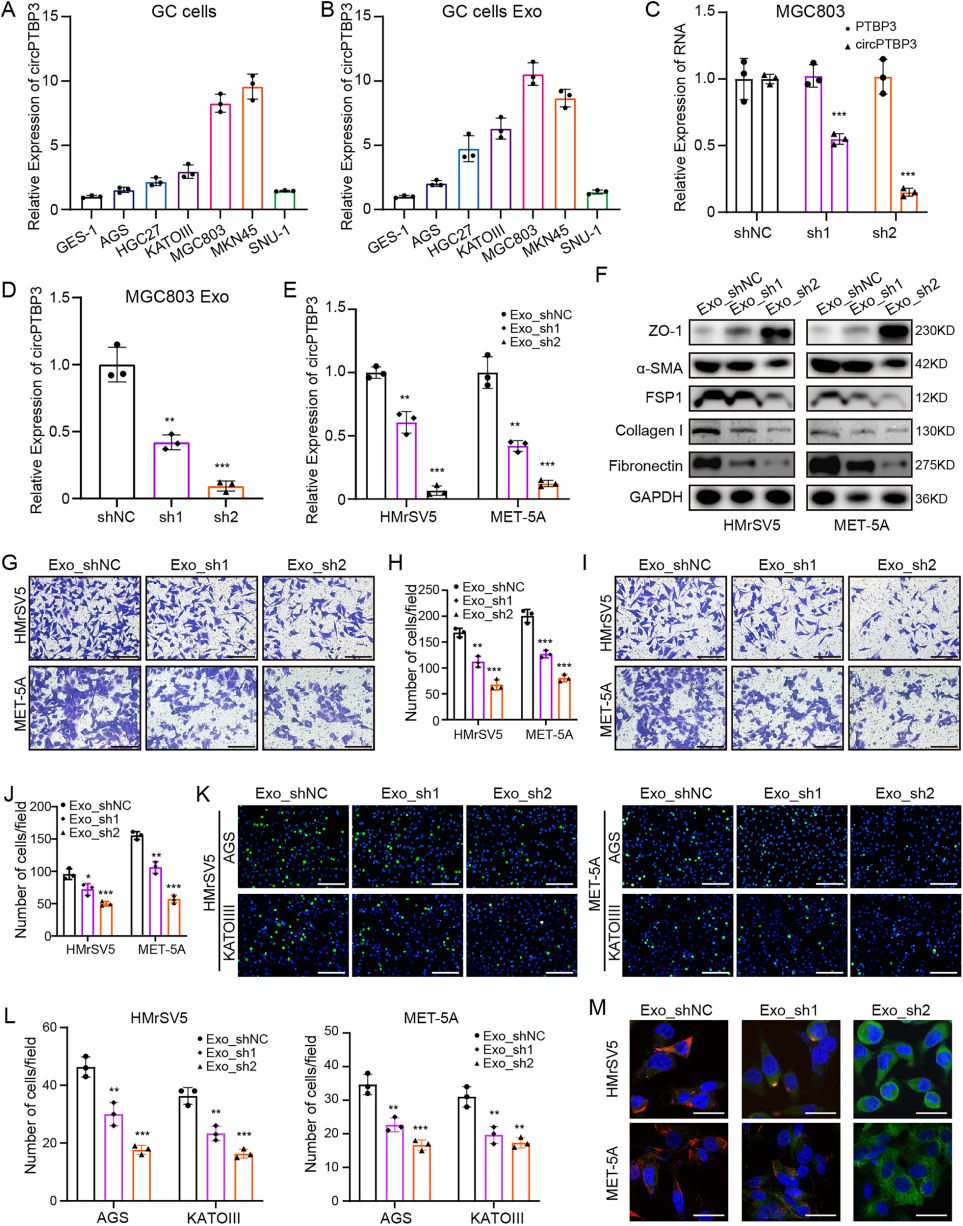
Exosomes extracted from circPTBP3 knockdown GC cells could inhibit the MMT of mesothelial cells in vitro. **A, B** The expression levels of circPTBP3 as well as exosomal circPTBP3 in different GC cells were determined by PCR analysis. **C** Construction lentivirus-mediated circPTBP3 knockdown MGC803 GC cells and its validation by PCR analysis. ShNC, control group; sh1, sh1 knockdown group; sh2, sh2 knockdown group. **D** Detection of circPTBP3 expression in exosomes from MGC803 cells in each group. **E** Detection of circPTBP3 expression in mesothelial cells treated by exosomes from MGC803 cells in each group. Exo_shNC, exosomes from MGC803 shNC group; exo_shs, exosomes from MGC803 sh1 group; Exo_sh2, exosomes from MGC803 sh2 group. **F** Detection of epithelial and mesenchymal proteins (ZO-1, α-SMA, FSP1, Collagen I, and Fibronectin) in mesothelial cells by western blot. **G**, **H** Observation and statistics of mesothelial cells migrating into the bottom surface of the chamber. Scale bars, 100 μm. **I, J** Observation and statistics of mesothelial cells invading the bottom surface of the chamber. Scale bars, 100 μm. **L** Observation and statistics of GC cells adhering to the mesothelial cell surface. Scale bars, 200 μm. **M** Immunofluorescence imaging of epithelial and mesenchymal markers of mesothelial cells. Blue, Hoechst33342, nucleus of mesothelial cells; green, ZO-1; red, fibronectin. Scale bars, 10 μm. (*p < 0.05; **p < 0.01; ***p < 0.001; ns, no significance).

Exosomes function as trucks carrying a variety of molecules for cell-to-cell communication. To elucidate the role of circPTBP3 in exosome-mediated MMT, we performed further experiments. First, we determined the expression levels of circPTBP3 in GES-1 and various GC cell lines, as well as in exosomes extracted from the cell culture supernatant, using RT-PCR (Fig. Fig 5A, B). We established PTBP3 stable knockdown MGC803 cells by lentivirus-packaged plasmids, however, silencing circPTBP3 did not affect the expression of linear PTBP3 (Fig. 5C). After downregulating circPTBP3 expression in MGC803 cells, the level of exosomal circPTBP3 was significantly reduced (Fig. 5D). Correspondingly, the expression of circPTBP3 in mesothelial cells was altered following treatment of these exosomes (Fig. 5E). Next, we evaluated the effects of exosomes extracted from circPTBP3 knockdown MGC803 cells (Exo_sh1 and_sh2) on mesothelial cells. Western blot analysis showed that treatment with circPTBP3-silenced exosomes increased ZO-1 protein expression while decreasing the expression of mesenchymal-related proteins, including α-SMA, FSP1, Collagen I, and Fibronectin (Fig. 5F). Additionally, these exosomes significantly suppressed the migratory, invasive and adhesive (adhesion to AGS or KATOIII cells) abilities of mesothelial cells, and altered ZO-1 and Fibronectin protein expressions in HMrSV5 and MET-5A cells by immunofluorescence assay (Fig. 5G-M). In particular, these exosomes inhibited the transformation of mesothelial cells into CAFs (Fig. S5).

Furthermore, we chose SNU-1 cells for circPTBP3 upregulation assay, and extracted exosomes to treat mesothelial cells (Fig. S6A-B). Oppositely, the similar experiments in vitro, including the western blot, cell migration, invasion, adhesion, and immunofluorescence analysis all strongly confirmed that exosomes derived from over-expressing circPTBP3 SNU-1 cells could obviously promote the MMT phenotype of mesothelial cells (Fig. S6C-K). Collectively, these data indicate that circPTBP3, secreted by GC cells and transported by exosomes, may act as a pivotal trigger of MMT in mesothelial cells.

### CircPTBP3 mediated MMT by enhancing SGK1 expression

**Fig. 6:**
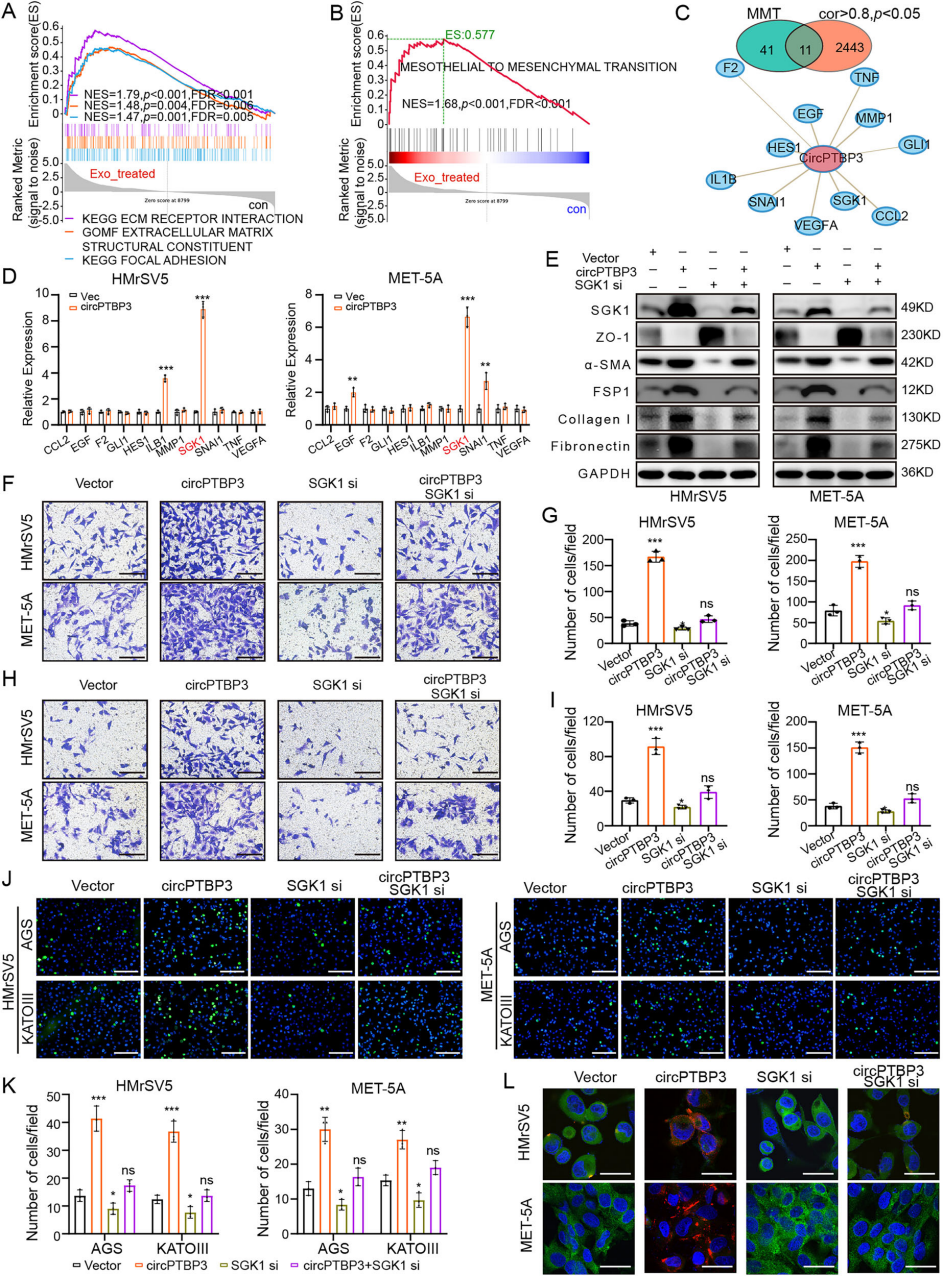
circPTBP3 mediated MMT by regulating SGK1 expression. **A** GSEA analysis of MMT-related gene sets by utilizing mRNA expression data in RNA-seq. **B** MMT gene set integration and GSEA analysis. **C** Venn analysis of circPTBP3 co-expression combined with MMT gene set. Pearson correlation coefficient >0.8 and *p* < 0.05 were considered as co-expressed genes of circPTBP3. **D** The expression of 11 candidate genes in mesothelial cells after circPTPB3 over-expression was detected by PCR analysis. Vec, control group; circPTBP3, circPTBP3 plasmid group. **E** Detection of epithelial and mesenchymal proteins (ZO-1, FSP1, α-SMA, Collagen I, and Fibronectin) in mesothelial cells by western blot. **F, G** Observation and statistics of mesothelial cells migrating into the bottom surface of the chamber. Scale bars, 100 μm. **H, I** Observation and statistics of mesothelial cells invading the bottom surface of the chamber. Scale bars, 100 μm. **J, K** Observation and statistics of GC cells adhering to the mesothelial cell surface. Scale bars, 200 μm. **L** Immunofluorescence imaging of epithelial and mesenchymal markers of mesothelial cells. Blue, Hoechst33342, nucleus of mesothelial cells; green, ZO-1; red, fibronectin. Scale bars, 10 μm. (*p < 0.05; **p < 0.01; ***p < 0.001; ns, no significance).

To further explore the possible downstream molecules of circPTBP3, we analyzed the mRNA data from our RNA-seq analysis (HMrSV5 cells after co-incubation with GC-derived exosomes). The results of Gene Set Enrichment Analysis (GSEA) showed that the terms “KEGG extracellular matrix receptor interaction,” “GOMF extracellular matrix structural constituent,” and “KEGG focal adhesion” were significantly enriched in mesothelial cells after the treatment of GC cell-derived exosomes (Fig. 6A). After searching PubMed for 2993 papers up to 2022 using the terms “mesothelial to mesenchymal transition” or “peritoneal fibrosis”, and integrating 52 key genes into an MMT geneset, GSEA analysis strongly indicated that MMT was significantly enriched following the exosome-treatment (Fig. 6B). A Venn analysis between 52 MMT-related genes and 2454 circPTBP3 co-expressing genes (correlation coefficient >0.8, *p* < 0.05) identified a total of 11 candidate genes that might be involved in circPTBP3-mediated MMT (Fig. 6C). Among these, the SGK1 mRNA expression was most significantly upregulated in HMrSV5 and MET-5A cells overexpressing circPTBP3, preliminarily suggesting that circPTBP3 might mediate MMT through SGK1 (Fig. 6D). To validate this hypothesis, a serious of rescue experiments were performed. Our data showed that silencing SGK1 expression significantly suppressed the phenotype of MMT in both HMrSV5 and MET-5A cells, including the MMT-related protein expressions, migratory, invasive, and adhesive (adhesion to AGS or KATOIII cells) abilities. More importantly, SGK1 knockdown in mesothelial cells could effectively offset the promoting effects of MMT caused by circPTBP3 over-expression (Fig. 6E-L and Fig. S7). These results strongly suggested that circPTBP3 potentially mediated the MMT of mesothelial cells by enhancing the SGK1 expression.

### CircPTBP3 enhanced SGK1 transcription by recruiting TFAP2B

**Fig. 7:**
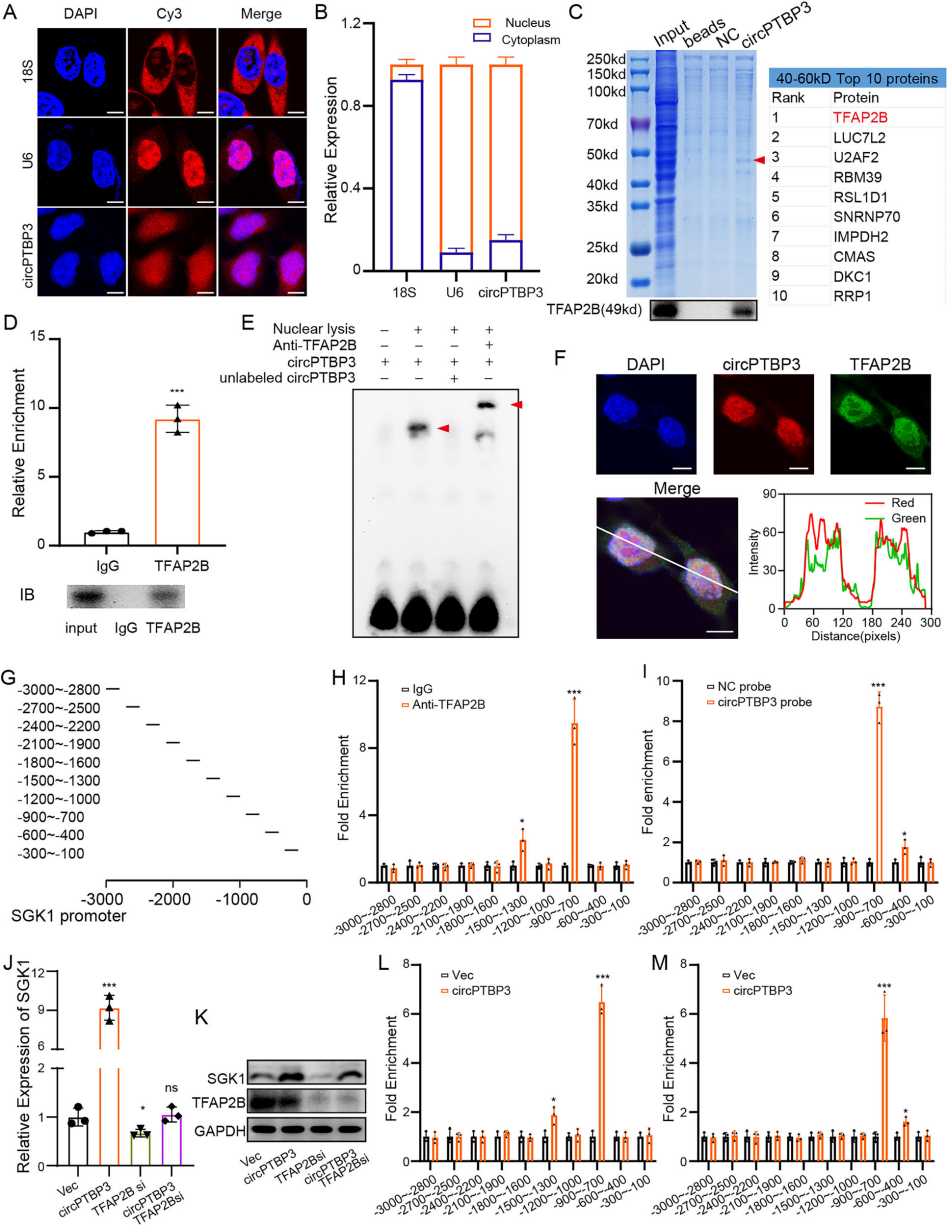
circPTBP3 enhanced SGK1 transcription by recruiting TFAP2B. **A** RNA FISH assay revealed the subcellular localization of circPTBP3 in HMrSV5 mesothelial cells. **B** Quantification of subcellular localization of circPTBP by nucleoplasmic separation combined with PCR analysis. **C** Gel staining of RNA pull-down assay and protein mass spectrometry. Beads, empty beads group; NC, NC probe group; circPTBP3, circPTBP3 probe group. Red arrows at the difference bands. Western blot assay revealed the specific binding of circPTBP3 to TFAP2B. **D** RIP assay confirmed the special binding of TFAP2B to circPTBP3.**(E** RNA EMSA confirmed the binding ability of the circPTBP3-specific probe to TFAP2B in vitro. The two red arrows indicated the trailing formed by circPTBP3-TFAP2B and circpTBP3-TFAP2B-anti-TFAP2B antibody complexes, respectively. **F** RNA FISH combined with immunofluorescence confirmed the co-localization of circPTBP3 and TFAP2B. **G** Schematic representation of primer construction for the SGK1 promoter. **H** ChIP assay was performed in circPTBP3 over-expressing HMrSV5 mesothelial cells. **I** ChIRP assay was performed in circPTBP3 over-expressing HMrSV5 mesothelial cells. **J, K** Detection of SGK1 mRNA and protein expressions in circPTBP3 over-expression or/and TFAP2B knockdown HMrSV5 cells. **L** ChIP assay was performed in HMrSV5 over-expression circPTBP3 group and the control group. **M** ChIRP assay was performed in HMrSV5 over-expression circPTBP3 group and the control group. (*p < 0.05; **p < 0.01; ***p < 0.001; ns, no significance).

We sought to elucidate the in-depth mechanisms between circPTBP3 and SGK1. Similar to U6, RNA FISH, and RT-PCR assays both revealed that circPTBP3 was predominantly localized in the nucleus of HMrSV5 cells (Fig. 7A-B). These results suggested that circPTBP3 might regulate the SGK1 expression by influencing its transcription through an RNA-binding protein (RBP)-driven mechanism, rather than by acting as miRNA sponges in the cytoplasm. To identify the RBPs interacting with circPTBP3, specific probes targeting the back-splice sites of circPTBP3 were designed, and an RNA pull-down assay was performed in HMrSV5 mesothelial cells. Gel imaging showed a distinct band near 50 kD for circPTBP3, and mass spectrometry (MS) analysis of proteins in the 40–60 kD range initially indicated that TFAP2B might be the most likely binding transcription factor (TF)(Fig. 7C and Table S3). Subsequent RIP and EMSA assays confirmed the binding of TFAP2B to circPTPB3 in the nucleus of mesothelial cells (Fig. 7D, E). RNA FISH combined with immunofluorescence demonstrated the co-localization of circPTBP3 and TFAP2B in the nucleus (Fig. 7F). To further explore the relationships among circPTB3, TFAP2B, and SGK1, the SGK1 promoter was divided into 10 fragments, and corresponding primers were designed, respectively (Fig. 7G). In HMrSV5 mesothelial cells overexpressing circPTBP3, ChIP assays showed that TFAP2B was enriched at specific SGK1 promoter sites (-700 ~ -900, -1300 ~ -1500), compared to the IgG control group (Fig. 7H). More importantly, ChIRP assays with circPTBP3 probes confirmed the enrichment of circPTBP3 at the SGK1 promoter regions (-400 to -600, -700 to -900) compared to the control probe group (–400 ~ –600, –700 ~ –900) (Fig. 7I). Subsequently, HMrSV5 cells were transfected with circPTBP3 over-expression plasmid as well as the siRNA fragments targeting TFAP2B. RT-qPCR and Western blot analyses demonstrated that TFAP2B knockdown significantly reversed the SGK1 upregulation caused by circPTBP3 overexpression (Fig. 7J). Finally, both TFAP2B and circPTBP3 were found to be significantly enriched at the SGK1 promoter regions in circPTBP3-overexpressing cells compared to control groups (Fig. 7L, M). Collectively, these in vitro data strongly suggest that circPTBP3 enhances SGK1 transcription by recruiting TFAP2B in mesothelial cells.

### Exosomal circPTBP3 promoted GCPM by inducing peritoneal fibrosis in vivo

**Fig. 8:**
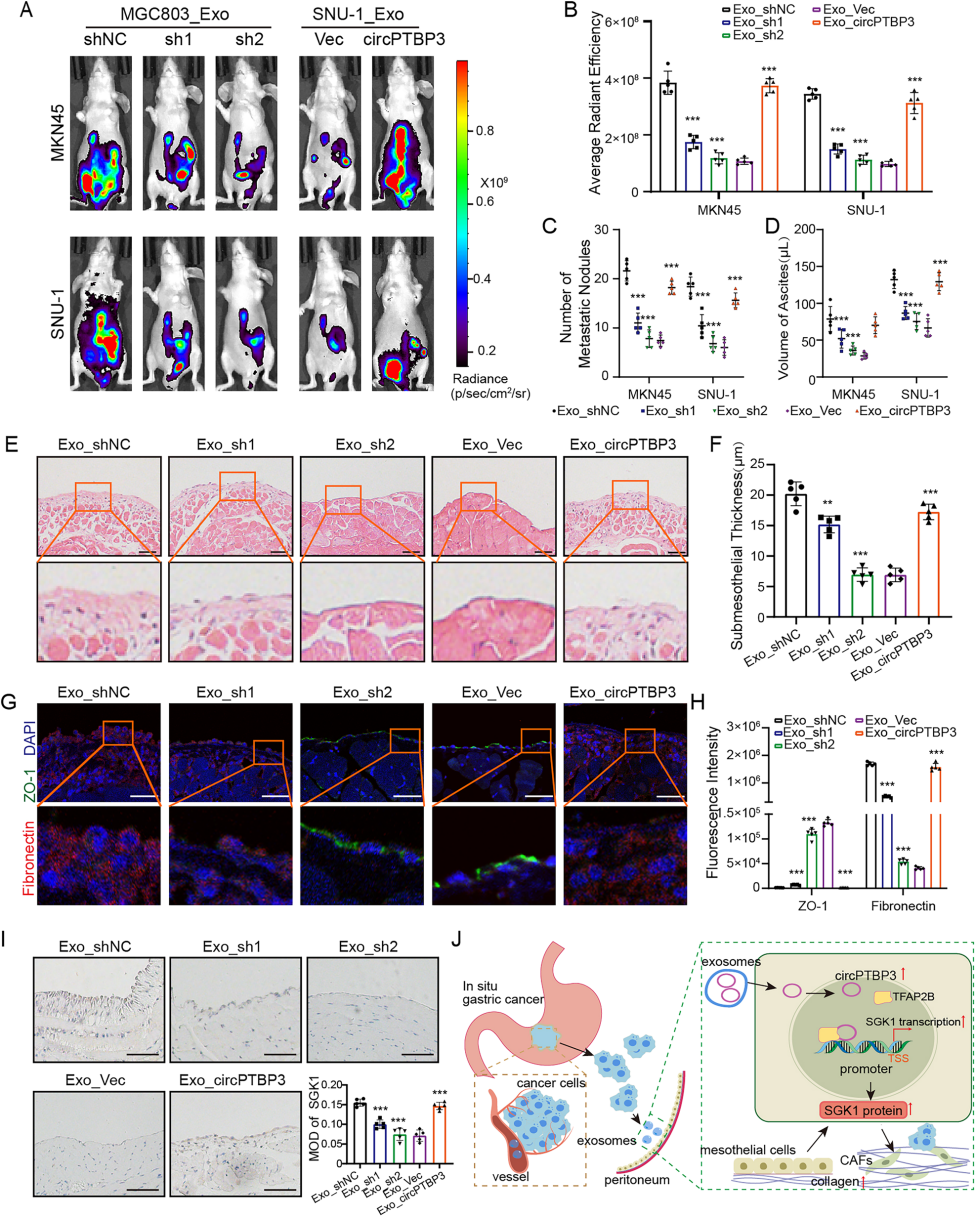
Exosomal circPTBP3 promoted GCPM by inducing peritoneal fibrosis in vivo. **A, B** IVIS imaging of peritoneal metastases after mice’s abdominal cavity was treated by exosomes from MGC803 cells and SNU-1 cells, and corresponding statistical analyses were presented. **C, D** Statistics of tumor nodules and ascites in the abdominal cavity of mice. **E, F** The peritoneum of mice was stained with HE and subcutaneous collagen thickness was compared. **G, H** Immunofluorescence staining and fluorescence intensity statistics of the peritoneal mesenchymal layer and collagen layer. Green, ZO-1; red, fibronectin. Scale bars, 50 μm. **I** Immunohistochemical staining and intensity statistics of SGK1 in peritoneal mesothelium. **J** Schematic of the mechanism by which circPTBP3 induced MMT in mesothelial cells. TSS, transcription start site. (*p < 0.05; **p < 0.01; ***p < 0.001; ns, no significance).

To validate the potential role of exosomal circPTBP3 in GCPM in vivo, we extracted exosomes from the circPTBP3 knockdown MGC803 cells and circPTBP3-overexpressing SNU-1 cells, respectively. Nude mice were intraperitoneally injected with MKN45 or SNU-1 GC cells one week after the treatment of the above-extracted exosomes, and the corresponding intraperitoneal tumor growth was observed by IVIS. Imaging results showed that exosomes extracted from circPTBP3 knockdown MGC803 cells significantly suppressed the peritoneal colonization of GC cells, while exosomes from circPTBP3-overexpressing SUN-1 cells markedly enhanced GCPM in mice (Fig. 8A, B). Tumor nodules in the abdominal cavity of mice were dissected, and ascites were collected, respectively. All these calculated results were positive and re-confirmed our previous observations by IVIS (Fig. 8C, D). Interestingly, circPTBP3 knockdown exosomes significantly reduced the thickness of peritoneal collagen, whereas exosomes with elevated circPTBP3 levels promoted peritoneal fibrosis in mice (Fig. 8E, F). In comparison with the control group, silencing circPTBP3 in MGC803 exosomes changed the ZO-1 and Fibronectin protein expressions by immunofluorescence assays. On the contrary, enhanced circPTPB3 expression in exosomes might lead to the opposite results (Fig. 8G, H). Characteristically, the expression of circPTBP3 in exosomes was positively correlated with the infiltration of CAFs derived from peritoneal mesothelial cells (Fig. S8). At last, we performed IHC staining of peritoneal samples using SGK1 antibody, and found that exosome-derived circPTPB3 could actually influence the SGK1 protein expressions in mice (Fig. 8I). Thus, we provided reliable evidence that exosomal circPTBP3 could promote GCPM by inducing peritoneal fibrosis in vivo.

## Discussion

Since their discovery in 1983, exosomes have been extensively investigated for their roles and mechanisms in intercellular communications, particularly in cancer research. A substantial body of evidence has suggested that exosomes can be transferred not only between cancer cells but also between cancer cells and stromal cells. The transferred materials include nucleic acids, lipids, proteins, and metabolites [27, 28]. As early as 2015, Tokuhisa et al. first evaluated the diagnostic potential of exosomal miRNA profiles in peritoneal fluid to predict the peritoneal dissemination of GC [29]. Since then, more tissue or cell-specific cargoes, including mRNAs, proteins, and miRNAs, were found to be accumulated and carried by exosomes to impact the progression and PM of GC by distinct mechanisms [30–34]. However, the roles of other exosomal components, such as circRNAs, have not been well characterized. In this study, we extracted exosomes from GC cells and observed that GC-derived exosomes could significantly enhance the GCPM by inducing peritoneal fibrosis in vivo. Meanwhile, the subsequent experiments in vitro also demonstrated that GC exosomes could promote the MMT of mesothelial cells. These data initially implied that GC-derived exosomes could facilitate PM by causing concurrent peritoneal fibrosis and MMT. Next, we extracted HMrsV5 cells after co-incubation with GC exosomes and performed the RNA-seq to screen the potential transported materials (circRNAs). Finally, the Venn analysis and validated data selected circPTBP3 as the most interesting molecule for our further studies.

CircPTBP3 was derived from the NM_001163793.2 transcript of PTBP3 locus on chromosome 9, and mainly back-spliced from exons 3, 4, and 5. Its parental gene PTPB3 is an essential RNA-binding protein with roles in RNA splicing, 3’ end processing, and translation. PTPB3 may function as an oncogenic or metastatic gene in GC progression [35, 36]. Other evidence also shows that PTPB3 plays a pivotal role in the regulation of GC differentiation [37]. For example, in 2020, PTPB3 was found to regulate the expression of Id1a and Id1b isoforms via alternative splicing, which might partly explain its inhibitory role in the differentiation of GC cells [38]. But until now, the roles of circPTPB3 (the spliced by-product of PTPB3) in cancers still seem mysterious. Subsequently, the clinical significance of circPTPB3 was systematically evaluated. Our data showed that both tissue and plasma exosomal circPTBP3 were predominantly over-expressed in GCPM patients. In comparison to the tissue circPTBP3 expression, the plasma exosomal circPTBP3 expression was more specific for diagnosing GCPM. Meanwhile, upregulated exosomal circPTBP3 were closely associated with some metastasis-related clinicopathologic features, including tumor differentiation, depth of invasion, lymphatic invasion, peritoneal metastasis, and TNM stage. A recent large-scale retrospective cohort study involving 13,447 cases found that in gastric cancer patients, Borrmann type IV, tumor invasion to T3-4, and lymph node metastasis were significantly associated with positive peritoneal lavage cytology [39]. Our study included a relatively high proportion of gastric cancer patients with peritoneal metastasis, which led to a greater number of confounding factors. However, after performing multivariate regression analysis to rebalance the various factors, the level of plasma exosomal circPTBP3 emerged as an independent prognostic factor for patients with gastric cancer peritoneal metastasis (GCPM). To our knowledge, this study is the first one to evaluate the diagnostic and prognostic role of circRNA in GCPM. Functionally, the data demonstrated that GC-derived circPTBP3 could significantly promote the MMT phenotype of mesothelial cells in vitro. SGK1 is an AGC protein kinase of the SGK family and can be identified and characterized as a tumor-promoting candidate in several malignancies, including breast cancer [40], colorectal cancer [41], non-small cell lung cancer [42], as well as GC [43, 44]. Growing evidence has also indicated that SGK1 does play a crucial role in tumor EMT and metastasis [45–48]. In this study, our results initially implied that SGK1 inhibition significantly attenuated the metastasis and MMT of mesothelial cells. Notably, SGK1 knockdown in mesothelial cells could effectively offset the promoting effects of MMT caused by exosomal circPTBP3 over-expression. These data strongly implied that exosomal circPTBP3 mediated MMT potentially by enhancing SGK1 expression.

Over the past decade, emerging evidence has revealed that many circRNAs are aberrantly expressed and exert biological functions through diverse mechanisms. CircRNAs primarily function as miRNA sponges, transcriptional regulators, or protein templates in GC [49–51]. Depending on their localization, nuclear circRNAs are mainly involved in gene transcription regulation [52]. For example, in 2019, circ-DONSON was highly expressed in GC tissues, and positively correlated with the advanced TNM stage and unfavorable prognosis. Dew to its location in the nucleus, circ-DONSON was found to recruit the NURF complex to the SOX4 promoter and activate its transcription [53]. circSLC22A23 was also localized in the nucleus of GC cells. A recent study from Wu et al. investigated the effects of circSLC22A23 on the GC progression and found that circSLC22A23 was able to initiate EGFR transcription through activation of HNRNPU [54]. Because of the intracellular location of circPTPB3 in mesothelial cells, we speculated that circPTBP3 might regulate the SGK1 expression potentially by influencing the transcription of SGK1 through an RBPs-driven mechanism. Fortunately, a series of experiments in vitro, including RNA FISH, RNA pull-down, RIP, EMSA, ChIP, and ChIRP assays, all confirmed that circPTPB3 could recruit TFAP2B to the SGK promoter sites in mesothelial cells, and initiate its transcription. What was more, the promoting role of exosomal circPTPB3 in GCPM by inducing peritoneal fibrosis had also been comprehensively illustrated in the intraperitoneal metastatic xenograft mice model (Fig. 8J).

To conclude, we proposed that GC-derived exosomes could significantly promote GCPM by causing concurrent peritoneal fibrosis and MMT in vivo and in vitro. RNA-seq as well as bioinformatics analyses were employed to screen the useful transported materials, and exosomal circPTBP3 was more specific for diagnosing GCPM. Up-regulated exosomal circPTBP3 expression closely correlated with a heavier tumor burden, and retained as an independent prognostic factor for GC survival. Mechanically, circPTPB3 could effectively recruit TFAP2B to the SGK1 promoter sites, and initiate its transcriptionin in mesothelial cells. In addition, it was also confirmed in vivo that exosomal circPTBP3 promoted GCPM by inducing peritoneal fibrosis. Taken together, our data revealed a novel MMT-promoting mechanism for exosomal circPTPB3 by interacting with TFAP2B and consequently enhancing the SGK1 expression in mesothelial cells.

## Materials and methods

### Cell culture

Human GC MGC803 cells were obtained from Beyotime Biotech (Shanghai, China). Other human GC cell lines (AGS, HGC27, MKN45, SNU-1, and KATOIII) and human mesothelial cell line MET-5A were purchased from Procell Life Science and Technology Co., Ltd. (Wuhan, China). The immortalized human gastric mucosal epithelial cell line GES-1, human GC cell line NCI-N87, and human mesothelial cell line HMrSV5 were maintained and reserved under a standard environment in our laboratory. All cells were maintained for growth at 37 °C in a humidified incubator with 5% CO2. KATOIII, MET-5A, and HMrSV5 cells were cultured in IMEM (Procell, Wuhan, China), M199 (Procell, Wuhan, China), and DMEM medium (Procell, Wuhan, China), respectively. The rest cell lines were cultured in RPMI-1640 medium (Invitrogen, Carlsbad, CA, USA). All medium was supplemented with 10% fetal bovine serum (Procell, Wuhan, China) and 1% penicillin/streptomycin.

### Exosome isolation, identification and labeling

GC cell-derived exosomes were purified by ultracentrifugation. Firstly, supernatants of GC cells cultured in exosome-free medium were subjected to differential centrifugation to remove cell debris and large particles. Secondly, exosomes were separated by ultracentrifugation (Beckman, USA) at 100,000 *g*, 70 min twice, and the precipitates were resuspended in PBS at the interval. Finally, exosomes were collected in PBS or stored at –80 °C. Human plasma exosomes were isolated with the exoEasy Maxi Kit (QIAGEN, Germany) according to the manufacturer’s instructions. Transmission electron microscopy (FEI TECNAI G2, USA) was used to examine the shape, size, and structure of exosomes, and nanoparticle tracking analysis (Malvern, UK) was applied for the quantified identification of exosome diameter. Exosome quantification was determined according to the protein concentration detected by the BCA kit (Beyotime Biotechnology, China). Exosome-related proteins were detected by western blot. Exosomes were stained with the green fluorescent dye PKH67 (Sigma-Aldrich, USA) as described in the instructions.

### Western blot

Protein samples were electrophoresed in 7.5% or 10% SDS-PAGE gels after quantification and denaturation and transferred to PVDF membranes by electrophoresis at 4 °C. The PVDF membranes were blocked and incubated sequentially with primary and secondary antibodies. Finally, enhanced chemiluminescence (ECL) imaging was applied to detect protein signals. The anti-CD9 antibody (mouse, monoclonal, Cat# 60232-1-Ig), anti-CD63 antibody (mouse, monoclonal, Cat# 67605-1-Ig), anti-TSG101 antibody (mouse, monoclonal, Cat# 67381-1-Ig), anti-ZO-1 antibody (rabbit, monoclonal, Cat# 21773-1-AP), anti-type I collagen (Collagen I) antibody (mouse, monoclonal, Cat# 66761-1-Ig), anti-fibronectin antibody (mouse, monoclonal, Cat# 66042-1-Ig), anti-FSP1/S100A4 antibody (mouse, monoclonal, Cat# 66489-1-Ig) and anti-SGK1 antibody (rabbit, polyclonal, Cat# 28454-1-AP) were all purchased from Proteintech (USA). The anti-alpha smooth muscle actin (α-SMA) antibody (rabbit, monoclonal, Cat# CY5027), anti-GAPDH antibody (mouse, monoclonal, Cat# AF2819) and anti-TFAP2B antibody (rabbit, polyclonal, Cat# 2509S) were obtained from Abways (China), Beyotime Biotech (China) and Cell Signaling Technology (USA), respectively. All the above antibodies were used at a 1:1000 dilution.

### In vivo assay

Male BALB/c nude mice at 4-weeks-old were purchased from Shanghai SLAC Laboratory Animal Co., Ltd. Animals were housed in SPF-grade standardized barrier environments. All animal experiments were approved by the Laboratory Animal Center of Soochow University (approval number: 202302A0168, 202302A0166) and followed the guidelines of AAALAC and IACUC.

To evaluate the role of exosomes in GCPM, mice were intraperitoneally injected with 100 μg of exosomes or PBS every two days for a week. Then 4 × 10^6^ luciferase-labeled MKN45 or SNU-1 (MKN45-Luc and SNU-1-Luc) cells were intraperitoneally injected into mice (5 mice per group) to establish the GC intraperitoneal xenograft model. An in vivo imaging system (IVIS) was applied to monitor the peritoneal dissemination of GC cells. Mice were sacrificed after 30 days, and the number of tumor nodules and ascites were quantified. The peritoneal metastatic tumor tissues and peritoneum were fixed and sectioned for hematoxylin-eosin (HE) staining or immunofluorescence (IF) staining. To examine the effects of exosomes on the peritoneum, mice were sacrificed directly after intraperitoneal injection of exosomes for a week. The peritoneum was fixed for HE staining or IF staining.

### Immunofluorescence (IF) staining

Peritoneal sections or cell samples were fixed and blocked, and incubated with primary antibodies overnight. Samples were washed and incubated with Cy3 or Alexa Fluor 488-labeled secondary antibodies. The final nuclei were stained with Hoechst33342 or DAPI. Images were taken by fluorescence microscopy. The anti-ZO-1 antibody (rabbit, monoclonal, Cat# 21773-1-AP), anti-FSP1/S100A4 antibody (mouse, monoclonal, Cat# 66489-1-Ig), anti-calretinin antibody (mouse, recombinant, Cat# 82811-1-RR) and anti-Fibronectin antibody (mouse, monoclonal, Cat# 66042-1-Ig) were purchased from Proteintech (USA). The anti-TFAP2B antibody (rabbit, polyclonal, Cat# 2509S) and anti-α-SMA antibody (rabbit, recombinant, Cat# CY1132) were obtained from Cell Signaling Technology (USA) and Abways (China), respectively. The primary and secondary antibodies were used at a 1:100 and 1:500 dilution, respectively.

### Transwell migration and invasion assays

Transwell assays were performed in 24-well plates and chambers (Coring, USA) were pretreated or not with Matrigel (BD, 356234). Mesothelial cells from each group were connected and resuspended in an FBS-free medium. After counting, the same number of cells were added to each upper chamber, and a medium supplemented with 10% FBS was added to the lower chamber. Upon completion, cells were photographed and counted under a microscope after being fixed and stained with crystal violet.

### Cell adhesion assay

Briefly, HMrSV5 or MET-5A mesothelial cells were used to prepare a monolayer of confluent cells in a glass-bottom dish. Before co-incubation, the mesothelial cell layer was stained with Hoechest33342 live-cell dye (Beyotime Biotechnology, China). Meanwhile, AGS or KATOIII GC cells were treated with Calcein AM (Beyotime Biotech, China). Then, 5 × 10^4^ GC cells were added to a glass-bottom dish and incubated with mesothelial cells for 30 min before free GC cells were washed away. The remaining GC cells were observed by fluorescence microscopy (100×).

### RNA sequencing

Total RNA was extracted from exosomes or PBS-treated HMrSV5 cells with Trizol reagent (Invitrogen, USA). The cDNA library was constructed after the removal of ribosomal RNA by OE Biotech (Shanghai, China), and high-throughput RNA sequencing was performed on the Illumina platform. After background correction and high-quality reads screening, the human genome (GRCh38) was used as the reference to obtain transcript data with HISAT2. CircRNAs were predicted by the authoritative software CIRI, and annotated based on the circBase database (http://circbase.org).

### Bioinformatics analysis

CircRNA expression profile in RNA sequencing data or GSE174237 dataset (https://www.ncbi.nlm.nih.gov/geo/query/acc.cgi?acc=GSE174237) were used for differential expression analysis. Differential expression analysis was performed by the R project (version 4.0.3, https://www.r-project.org) with the DEseq2 package. Fold change (FC) > 2 and *p-value* < 0.05 were used as the threshold to screen differentially expressed circRNAs. Heat maps and volcano maps were drawn via the ggplot2 package. Gene Set Enrichment Analysis (GSEA) was performed according to the instructions on the official website (https://www.gsea-msigdb.org/gsea). Co-expression analysis was performed based on Pearson correlation coefficient (Pearson r) >0.8 and *p-value* < 0.05.

### Tissue specimens

We collected the primary tumor tissues and adjacent mucosa tissues from 110 gastric cancer patients who visited the First Affiliated Hospital of Soochow University from 2011 to 2021. Among them, 80 patients had no peritoneal metastasis (NPM) confirmed by Ct and abdominal exploration, and 30 patients had PM. These patients did not receive neoadjuvant chemotherapy before surgery, and followed up regularly after surgery. In addition, plasma samples were collected from these GC patients before surgery and 30 healthy volunteers. Tissue RNA and plasma exosomes were extracted and frozen in liquid nitrogen. Informed consents were obtained from all participants, and the study complied with the principles described in the Declaration of Helsinki. In addition, this study was approved by the Ethics Committee of The First Affiliated Hospital of Soochow University (approval number: #2022164).

### Reverse transcription polymerase chain reaction (RT-PCR) and quantitative real-time PCR (qRT-PCR)

Nuclear and cytoplasmic fractions were isolated with the PARIS™ Kit (Invitrogen, USA), and the RNA of the corresponding fractions was isolated. The total RNA of tissues, cells, and exosomes was extracted by Trizol reagent (Invitrogen, USA). RNA was digested with RNase R at 3U/µg at 37 °C for 30 min. The reverse transcription system was configured according to the 5× All-In-One MasterMix (ABMgood, USA) and programmed to produce cDNA. The cDNAs were then collected for PCR or qRT-PCR. PCR was performed after mixing cDNA, primers, and Taq DNA polymerase, and the products were electrophoresed in 2% agarose gels. The qRT-PCR system was configured according to the SYBR qPCR kit (Novoprotein, China) and programmed for amplification by an ABI ViiA 7 instrument (Applied Biosystems, USA). The 2^-ΔΔCt^ value was calculated for standardized quantification of circRNAs and mRNAs. All primers were listed in Table S4.

### Plasmid and small interfering RNAs (siRNAs) construction, and stable transfection

Plasmids and siRNAs were designed and synthesized at Vigene Biosciences Vigene Biosciences (Shandong, China). To construct the circPTBP3 expression plasmid, the full-length sequence of circPTBP3 was inserted into pLV-Cir-circPTBP3, and the mock vector without the circPTBP3 sequence was used as a control. Plasmid and siRNA transfection was performed using Lipofectamine 3000 (Invitrogen, CA, USA), as described in the instructions. In stable cell preparation, green fluorescent protein and puromycin resistance genes were used to synthesize shRNA lentiviruses. Puromycin was used to screen successfully infected cells at 48 h post-infection while fluorescence intensity was measured. RT-qPCR was utilized to calculate over-expression and silencing efficiency.

### RNA fluorescence in situ hybridization (RNA FISH)

To identify the location of circPTBP3, specific probes were designed based on the back-splice site by Ribobio (China). HMrsV5 mesothelial cells were seeded on glass-bottom dishes overnight and then fixed. Cell samples were treated with 0.5% triton-100 and blocked with a prehybridization solution. At the end of blocking, the circPTBP3 probe, U6, and 18S probe were re-suspended by hybridization solution and overlaid on cell samples in a wet cassette overnight at 37 °C. After washing, nuclei were stained with DAPI. The final images were observed under a fluorescence microscope (600×).

### RNA pull-down assay and mass spectrometry (MS)

Biotin-labeled probes were synthesized by GenePharma (Shanghai, China) which specially targeted the backsplice site of circPTBP3, and an oligonucleotide probe was used as a control. HMrSV5 mesothelial cell lysates were mixed with the probe and incubated at room temperature for the appropriate time. HMrSV5 mesothelial cell lysates were mixed with probes and incubated appropriately to form probe-circpTBP3 complexes. The streptavidin-labeled magnetic beads were then added to the lysate to precipitate circPTBP3. Finally, circpTBP3-bound proteins were purified for SDS-PAGE gel electrophoresis and MS analysis.

### RNA binding protein immunoprecipitation (RIP) assay

RIP assay was performed to determine the binding ability of TFAP2B to circPTBP3 with a Magna RIP kit (Millipore Magna, USA). Briefly, anti-TFAP2B antibodies (rabbit, polyclonal, Cat# 2509S) or control rabbit IgG antibodies were conjugated with magnetic beads, and lysates of HMrSV5 mesothelial cells over-expressing circPTBP3 were incubated with magnetic beads. Finally, the RNA and protein were purified for RT-qPCR and western blot, respectively.

### RNA electrophoretic mobility shift assay (EMSA)

EMSA was performed to detect the binding ability of circPTBP3 to TFAP2B in vitro. Briefly, circPTBP3-specific EMSA probes were designed based on the back-splice site with or without biotin labeling. Nuclear lysates were obtained by isolation with the kit described above. After anti-ribonuclease treatment for all instruments, 4% PAGE gels were prepared. The four reaction systems were prepared according to the instructions of the chemiluminescence EMSA kit (Beyotime Biotech, China) and incubated at room temperature for 25 min. The components of the reaction system were separated after the first electrophoresis, and the probe was transferred to a positively charged nylon membrane by the second electrophoresis. The nylon membranes were cross-linked with appropriate UV light and incubated with HRP-labeled streptavidin. Finally, the chemiluminescence method was used to obtain probe migration images.

### Chromatin Immunoprecipitation (ChIP)

ChIP Assay Kit (Beyotime Biotech, China) was used for the ChIP assay. HMrSV5 cells were cross-linked with 1% formaldehyde for 10 min. After termination of cross-linking, cell lysates were prepared to break DNA into 200 to 500 bp length fragments using ultrasound (Bioruptor). Cell lysates were incubated with anti-TFAP2B antibody (rabbit, polyclonal, Cat# 2509S) or rabbit Ig antibody, and then protein A + G agarose was added to precipitate DNA-protein complexes. After the DNA from the complexes was purified, RT-qPCR was performed for identification of the SGK1 promoter. The specific primers were designed to fragment the SGK1 promoter into ~200 bp segments (Table S4).

### Chromatin Isolation by RNA Purification (ChIRP)

In brief, after glutaraldehyde cross-linking and glycine termination, HMrsV5 mesothelial cells were harvested, and cell lysates were prepared. DNA was ultrasonically broken into 200 to 500 bp lengths. Biotin-labeled circPTBP3 probe and control probe were added to the lysate and incubated at 37 °C for 4 h. Streptavidin-labeled magnetic beads were added and used to precipitate the chromatin fragments, to which the probe was bound. DNA was purified for RT-PCR.

### Immunohistochemistry (IHC)

IHC was used to detect the expression of SGK1 in peritoneal tissues. Briefly, peritoneal paraffin sections were deparaffinized with xylene, rehydrated with gradient ethanol, and boiled in sodium citrate solution. Sections were blocked and incubated with SGK1 antibody or rabbit IgG antibody at 4 °C overnight, followed by incubation with HRP-conjugated secondary antibodies. Finally, the color reaction of peritoneal sections was carried out according to the instructions with the DAB Horseradish Peroxidase Color Development Kit (Beyotime Biotech, China).

### Statistical analysis

Statistical analysis was performed by GraphPad Prism 9.0 (USA) and SPSS 26.0 (USA). Continuous data were expressed as mean ± standard deviation (SD), and the comparison of continuous data between the two groups was performed with the g two-tailed Student’s *t*-test. The chi-square test was used for comparison of categorical data. The receiver operating characteristic (ROC) curve was utilized to demonstrate the diagnostic ability of the classification model. The log-rank test was calculated to compare survival differences between the two groups, and the COX proportional hazard regression model was used to evaluate the effect of multivariate factors on survival. *p* < 0.05 was considered signifcant (*p < 0.05; **p < 0.01; *** *p* < 0.001), while ns was considered non-signifcant.

## Supplementary information

Original Western Blots

Supplementary Figures and Tables

## Data Availability

The RNA-seq dataset supporting the conclusions of this article is available in Science Data Bank (ScienceDB, https://www.scidb.cn/en, DIO number 10.57760/sciencedb.08330). The other resources used in this study are available from the corresponding authors upon reasonable request.

## References

[1] Koemans WJ, Lurvink RJ, Grootscholten C, Verhoeven RHA, de Hingh IH, van Sandick JW. Synchronous peritoneal metastases of gastric cancer origin: incidence, treatment and survival of a nationwide Dutch cohort. Gastric Cancer. 2021;24:800–9. 33495964 10.1007/s10120-021-01160-1

[2] Fujitani K, Yang HK, Mizusawa J, Kim YW, Terashima M, Han SU, et al. Gastrectomy plus chemotherapy versus chemotherapy alone for advanced gastric cancer with a single non-curable factor (REGATTA): a phase 3, randomised controlled trial. Lancet Oncol. 2016;17:309–18. 26822397 10.1016/S1470-2045(15)00553-7

[3] Foster JM, Zhang CM, Rehman S, Sharma P, Alexander HR. The contemporary management of peritoneal metastasis: A journey from the cold past of treatment futility to a warm present and a bright future. CA-Cancer J Clin. 2023;73:49–71. 35969103 10.3322/caac.21749

[4] Bootsma S, Bijlsma MF, Vermeulen L. The molecular biology of peritoneal metastatic disease. EMBO Mol Med. 2023;15:11. 10.15252/emmm.202215914 PMC9994485 36700339

[5] Cortés-Guiral D, Hübner M, Alyami M, Bhatt A, Ceelen W, Glehen O, et al. Primary and metastatic peritoneal surface malignancies. Nat Rev Dis Prim. 2021;7:23. 34916522 10.1038/s41572-021-00326-6

[6] Roth L, Russo L, Ulugoel S, dos, Santos RF, Breuer E, et al. Peritoneal Metastasis: Current Status and Treatment Options. Cancers. 2022;14:14. 10.3390/cancers14010060 PMC8750973 35008221

[7] Mei SS, Chen X, Wang K, Chen YX. Tumor microenvironment in ovarian cancer peritoneal metastasis. Cancer Cell Int. 2023;23:13. 36698173 10.1186/s12935-023-02854-5 PMC9875479

[8] van Baal J, van de Vijver KK, Nieuwland R, van Noorden CJF, van Driel WJ, Sturk A, et al. The histophysiology and pathophysiology of the peritoneum. Tissue Cell. 2017;49:95–105. 27890350 10.1016/j.tice.2016.11.004

[9] Mutsaers SE. Mesothelial cells: Their structure, function and role in serosal repair. Respirology. 2002;7:171–91. 12153683 10.1046/j.1440-1843.2002.00404.x

[10] Ramos C, Gerakopoulos V, Oehler R. Metastasis-associated fibroblasts in peritoneal surface malignancies. Br J Cancer. 2024;131:407–19. 10.1038/s41416-024-02717-4 PMC11300623 38783165

[11] Guo TC, Xu JF. Cancer-associated fibroblasts: a versatile mediator in tumor progression, metastasis, and targeted therapy. Cancer Metastasis Rev. 2024;43:1095–116. 10.1007/s10555-024-10186-7 PMC11300527 38602594

[12] Deng G, Qu JL, Zhang Y, Che XF, Cheng Y, Fan YB, et al. Gastric cancer-derived exosomes promote peritoneal metastasis by destroying the mesothelial barrier. FEBS Lett. 2017;591:2167–79. 28643334 10.1002/1873-3468.12722

[13] Liu BQ, Shen H, He J, Jin BH, Tian YS, Li WQ, et al. Cytoskeleton remodeling mediated by circRNA-YBX1 phase separation suppresses the metastasis of liver cancer. Proc Natl Acad Sci USA. 2023;120:12. 10.1073/pnas.2220296120 PMC10372620 37459535

[14] Wan DW, Wang ST, Xu ZH, Zan XQ, Liu F, Han Y, et al. PRKAR2A-derived circular RNAs promote the malignant transformation of colitis and distinguish patients with colitis-associated colorectal cancer. Clin Transl Med. 2022;12:20. 10.1002/ctm2.683 PMC8858608 35184406

[15] Lun J, Zhang YY, Yu MC, Zhai WX, Zhu L, Liu HZ, et al. Circular RNA circHIPK2 inhibits colon cancer cells through miR-373-3p/ RGMA axis. Cancer Lett. 2024;593:13. 10.1016/j.canlet.2024.216957 38762192

[16] Ding LF, Wang RY, Zheng QM, Shen DY, Wang H, Lu ZY, et al. circPDE5A regulates prostate cancer metastasis via controlling WTAP-dependent N6-methyladenisine methylation of EIF3C mRNA. J Exp Clin Cancer Res. 2022;41:19. 35650605 10.1186/s13046-022-02391-5 PMC9161465

[17] Yuan Y, Zhang XJ, Du KN, Zhu XH, Chang SS, Chen Y, et al. Circ_CEA promotes the interaction between the p53 and cyclin-dependent kinases 1 as a scaffold to inhibit the apoptosis of gastric cancer. Cell Death Dis. 2022;13:15. 10.1038/s41419-022-05254-1 PMC9515085 36167685

[18] Xia YW, Jiang TL, Li Y, Gu C, Lv JL, Lu C, et al. circVAPA-rich small extracellular vesicles derived from gastric cancer promote neural invasion by inhibiting SLIT2 expression in neuronal cells. Cancer Lett. 2024;592:17. 10.1016/j.canlet.2024.216926 38714291

[19] Fang L, Lv JL, Xuan Z, Li BW, Li Z, He ZY, et al. Circular CPM promotes chemoresistance of gastric cancer via activating PRKAA2-mediated autophagy. Clin Transl Med. 2022;12:18. 10.1002/ctm2.708 PMC8787023 35075806

[20] Zhang F, Jiang JJ, Qian H, Yan YM, Xu WR. Exosomal circRNA: emerging insights into cancer progression and clinical application potential. J Hematol Oncol. 2023;16:25. 37365670 10.1186/s13045-023-01452-2 PMC10294326

[21] Zhang CG, Wei GX, Zhu XX, Chen X, Ma XX, Hu P, et al. Exosome-Delivered circSTAU2 Inhibits the Progression of Gastric Cancer by Targeting the miR-589/CAPZA1 Axis. Int J Nanomed. 2023;18:127–42. 10.2147/IJN.S391872 PMC9832994 36643863

[22] Li X, Lin YL, Shao JK, Wu XJ, Li X, Yao H, et al. Plasma exosomal hsa_circ_0079439 as a novel biomarker for early detection of gastric cancer. World J Gastroenterol. 2023;29:3482–96. 37389236 10.3748/wjg.v29.i22.3482 PMC10303519

[23] Zheng XM, Xiao HW, Liu XX, Huang T, Deng CW. Exosomal circKIAA1797 Regulates Cell Progression and Glycolysis by Targeting miR-4429/PBX3 Pathway in Gastric Cancer. Biochem Genet. 2024;62:1762–78. 37730964 10.1007/s10528-023-10529-z

[24] Zhang YX, Xie WR, Zheng WH, Qian XY, Deng CW. Exosome-mediated circGMPS facilitates the development of gastric cancer cells through miR-144-3p/PUM1. Cytotechnology. 2024;76:53–68. 38304630 10.1007/s10616-023-00597-9 PMC10828494

[25] Deng CC, Huo MY, Chu HW, Zhuang XM, Deng GF, Li WC, et al. Exosome circATP8A1 induces macrophage M2 polarization by regulating the miR-1-3p/STAT6 axis to promote gastric cancer progression. Mol Cancer. 2024;23:19. 38459596 10.1186/s12943-024-01966-4 PMC10921793

[26] Wang Y, Zou R, Li DK, Gao XK, Lu XJ. Exosomal circSTRBP from cancer cells facilitates gastric cancer progression via regulating miR-1294/miR-593-3p/E2F2 axis. J Cell Mol Med. 2024;28:16. 10.1111/jcmm.18217 PMC10960172 38520208

[27] Kalluri R, LeBleu VS. The biology, function, and biomedical applications of exosomes. Science. 2020;367:640. 10.1126/science.aau6977 PMC7717626 32029601

[28] Wortzel I, Dror S, Kenific CM, Lyden D. Exosome-Mediated Metastasis: Communication from a Distance. Dev Cell. 2019;49:347–60. 31063754 10.1016/j.devcel.2019.04.011

[29] Tokuhisa M, Ichikawa Y, Kosaka N, Ochiya T, Yashiro M, Hirakawa K, et al. Exosomal miRNAs from Peritoneum Lavage Fluid as Potential Prognostic Biomarkers of Peritoneal Metastasis in Gastric Cancer. PLoS One. 2015;10:13. 10.1371/journal.pone.0130472 PMC4514651 26208314

[30] Luo JX, Jiang LX, He CY, Shi MM, Yang ZY, Shi M, et al. Exosomal hsa-let-7g-3p and hsa-miR-10395-3p derived from peritoneal lavage predict peritoneal metastasis and the efficacy of neoadjuvant intraperitoneal and systemic chemotherapy in patients with gastric cancer. Gastric Cancer. 2023;26:364–78. 36738390 10.1007/s10120-023-01368-3

[31] Makinoya M, Miyatani K, Matsumi Y, Sakano Y, Shimizu S, Shishido Y, et al. Exosomal miR-493 suppresses MAD2L1 and induces chemoresistance to intraperitoneal paclitaxel therapy in gastric cancer patients with peritoneal metastasis. Sci Rep. 2024;14:12. 38698201 10.1038/s41598-024-60967-x PMC11065888

[32] Zhu AK, Shan YQ, Zhang J, Liu XC, Ying RC, Kong WC. Exosomal NNMT from peritoneum lavage fluid promotes peritoneal metastasis in gastric cancer. Kaohsiung J Med Sci. 2021;37:305–13. 33508890 10.1002/kjm2.12334 PMC11896370

[33] Zhou CF, Qiao CT, Ji J, Xi WQ, Jiang JL, Guo LT, et al. Plasma Exosome Proteins ILK1 and CD14 Correlated with Organ-Specific Metastasis in Advanced Gastric Cancer Patients. Cancers. 2023;15:3986. 37568802 10.3390/cancers15153986 PMC10417498

[34] Chen YY, Cai GX, Jiang JJ, He C, Chen YR, Ding YF, et al. Proteomic profiling of gastric cancer with peritoneal metastasis identifies a protein signature associated with immune microenvironment and patient outcome. Gastric Cancer. 2023;26:504–16. 36930369 10.1007/s10120-023-01379-0 PMC10284991

[35] Lin GR, Chen WR, Zheng PH, Chen WS, Cai GY. Circular RNA circ_0006089 promotes the progression of gastric cancer by regulating the miR-143-3p/PTBP3 axis and PI3K/AKT signaling pathway. J Dig Dis. 2022;23:376–87. 35844201 10.1111/1751-2980.13116

[36] Liang X, Chen WX, Shi HY, Gu XY, Li YQ, Qi YX, et al. PTBP3 contributes to the metastasis of gastric cancer by mediating CAV1 alternative splicing. Cell Death Dis. 2018;9:13. 29752441 10.1038/s41419-018-0608-8 PMC5948206

[37] Liang X, Shi HY, Yang LY, Qiu C, Lin SC, Qi YX, et al. Inhibition of polypyrimidine tract-binding protein 3 induces apoptosis and cell cycle arrest, and enhances the cytotoxicity of 5-fluorouracil in gastric cancer cells. Br J Cancer. 2017;116:903–11. 28222070 10.1038/bjc.2017.32 PMC5379144

[38] Chen B, Chen WX, Mu XY, Yang LY, Gu XY, Zhao AG, et al. PTBP3 Induced Inhibition of Differentiation of Gastric Cancer Cells Through Alternative Splicing of Id1. Front Oncol. 2020;10:12. 32974175 10.3389/fonc.2020.01477 PMC7461954

[39] Kim S, Lee HH, Song KY, Seo HS. Peritoneal Washing Cytology Positivity in Gastric Cancer: Role of Lymph Node Metastasis as a Risk Factor. J Gastric Cancer. 2024;24:185–98. 38575511 10.5230/jgc.2024.24.e14 PMC10995825

[40] Hall BA, Kim TY, Skor MN, Conzen SD. Serum and glucocorticoid-regulated kinase 1 (SGK1) activation in breast cancer: requirement for mTORC1 activity associates with ER-alpha expression. Breast Cancer Res Treat. 2012;135:469–79. 22842983 10.1007/s10549-012-2161-y PMC3891577

[41] Lee LYW, Woolley C, Starkey T, Biswas S, Mirshahi T, Bardella C, et al. Serum- and Glucocorticoid-induced Kinase Sgk1 Directly Promotes the Differentiation of Colorectal Cancer Cells and Restrains Metastasis. Clin Cancer Res. 2019;25:629–40. 30322876 10.1158/1078-0432.CCR-18-1033 PMC6339518

[42] Yu XB, Lin Q, Qin X, Ruan Z, Zhou JH, Lin ZF, et al. Serum and glucocorticoid kinase 1 promoted the growth and migration of non-small cell lung cancer cells. Gene. 2016;576:339–46. 26548813 10.1016/j.gene.2015.10.072

[43] Zhang JS, Lv W, Liu YG, Fu WH, Chen BS, Ma QT, et al. Knockdown of Serum- and Glucocorticoid-Regulated Kinase 1 Enhances Cisplatin Sensitivity of Gastric Cancer Through Suppressing the Nuclear Factor Kappa-B Signaling Pathway. Balk Med J. 2021;38:331. 10.5152/balkanmedj.2021.21114 PMC8880965 34860160

[44] Gu ZY, Wang LP, Yao XH, Long Q, Lee KP, Li JY, et al. ClC-3/SGK1 regulatory axis enhances the olaparib-induced antitumor effect in human stomach adenocarcinoma. Cell Death Dis. 2020;11:16. 33093458 10.1038/s41419-020-03107-3 PMC7583252

[45] Cheng JZ, Truong LD, Wu XQ, Kuhl D, Lang F, Du J. Serum- and glucocorticoid-regulated kinase 1 is upregulated following unilateral ureteral obstruction causing epithelial-mesenchymal transition. Kidney Int. 2010;78:668–78. 20631674 10.1038/ki.2010.214 PMC3935313

[46] Gu X, Meng HY, Peng CY, Lin SY, Li BH, Zhao L, et al. Inflammasome activation and metabolic remodelling in p16-positive aging cells aggravates high-fat diet-induced lung fibrosis by inhibiting NEDD4L-mediated K48-polyubiquitin-dependent degradation of SGK1. Clin Transl Med 2023;13: 23. 10.1002/ctm2.1308 PMC10285269 37345264

[47] Lee SG, Kim D, Lee JJ, Lee HJ, Moon RK, Lee YJ, et al. Dapagliflozin attenuates diabetes-induced diastolic dysfunction and cardiac fibrosis by regulating SGK1 signaling. BMC Med. 2022;20:12. 36068525 10.1186/s12916-022-02485-z PMC9450279

[48] Zhang L, Liu J, Liu YC, Xu YG, Zhao XF, Qian J, et al. Fluvastatin inhibits the expression of fibronectin in human peritoneal mesothelial cells induced by high-glucose peritoneal dialysis solution via SGK1 pathway. Clin Exp Nephrol. 2015;19:336–42. 24942605 10.1007/s10157-014-0991-0

[49] Rao DA, Yu CP, Sheng JQ, Lv EJ, Huang WJ. The Emerging Roles of circFOXO3 in Cancer. Front Cell Dev Biol. 2021;9:9. 10.3389/fcell.2021.659417 PMC8213346 34150756

[50] Wang LY, Long HY, Zheng QH, Bo XT, Xiao XH, Li B. Circular RNA circRHOT1 promotes hepatocellular carcinoma progression by initiation of NR2F6 expression. Mol Cancer. 2019;18:12. 31324186 10.1186/s12943-019-1046-7 PMC6639939

[51] Chen CK, Cheng R, Demeter J, Chen J, Weingarten-Gabbay S, Jiang LH, et al. Structured elements drive extensive circular RNA translation. Mol Cell. 2021;81:4300. 34437836 10.1016/j.molcel.2021.07.042 PMC8567535

[52] Liu CX, Chen LL. Circular RNAs: Characterization, cellular roles, and applications. Cell. 2022;185:2016–34. 35584701 10.1016/j.cell.2022.04.021

[53] Ding LX, Zhao YY, Dang SW, Wang Y, Li XL, Yu XT, et al. Circular RNA circ-DONSON facilitates gastric cancer growth and invasion via NURF complex dependent activation o transcription factor SOX4. Mol Cancer. 2019;18:11. 30922402 10.1186/s12943-019-1006-2 PMC6437893

[54] Wu XX, Cao CL, Li Z, Xie YY, Zhang SS, Sun WL, et al. Circular RNA CircSLC22A23 Promotes Gastric Cancer Progression by Activating HNRNPU Expression. Dig Dis Sci. 2024;69:1200–13. 38400886 10.1007/s10620-024-08291-2

