# Supplementary material for: Tumor exosomal circPTBP3 drives gastric cancer peritoneal metastasis via mesothelial-mesenchymal transition
Source: Cell Death Dis. 2025 Jun 11;16(1):444. doi: 10.1038/s41419-025-07749-z (PMC12159144; doi:10.1038/s41419-025-07749-z)
Supplement: Supplementary file 1 — Original Western Blots [file 41419_2025_7749_MOESM1_ESM.pdf]

Figure 1C

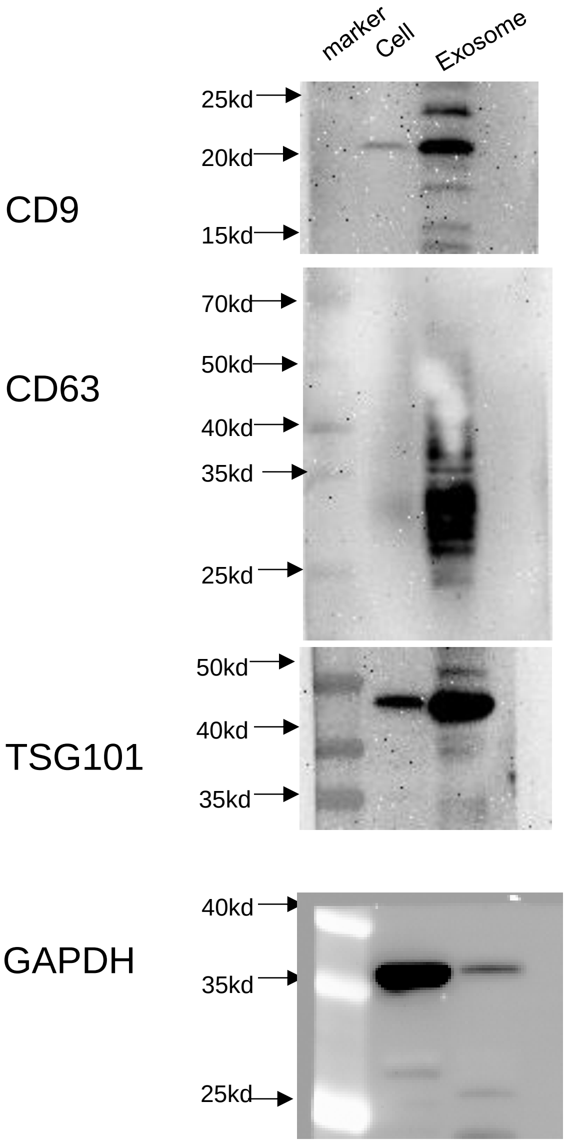
marker
Cell
Exosome
CD9
25kd
20kd
15kd
CD63
70kd
50kd
40kd
35kd
25kd
TSG101
50kd
40kd
35kd
GAPDH
40kd
35kd
25kd

Figure 2C

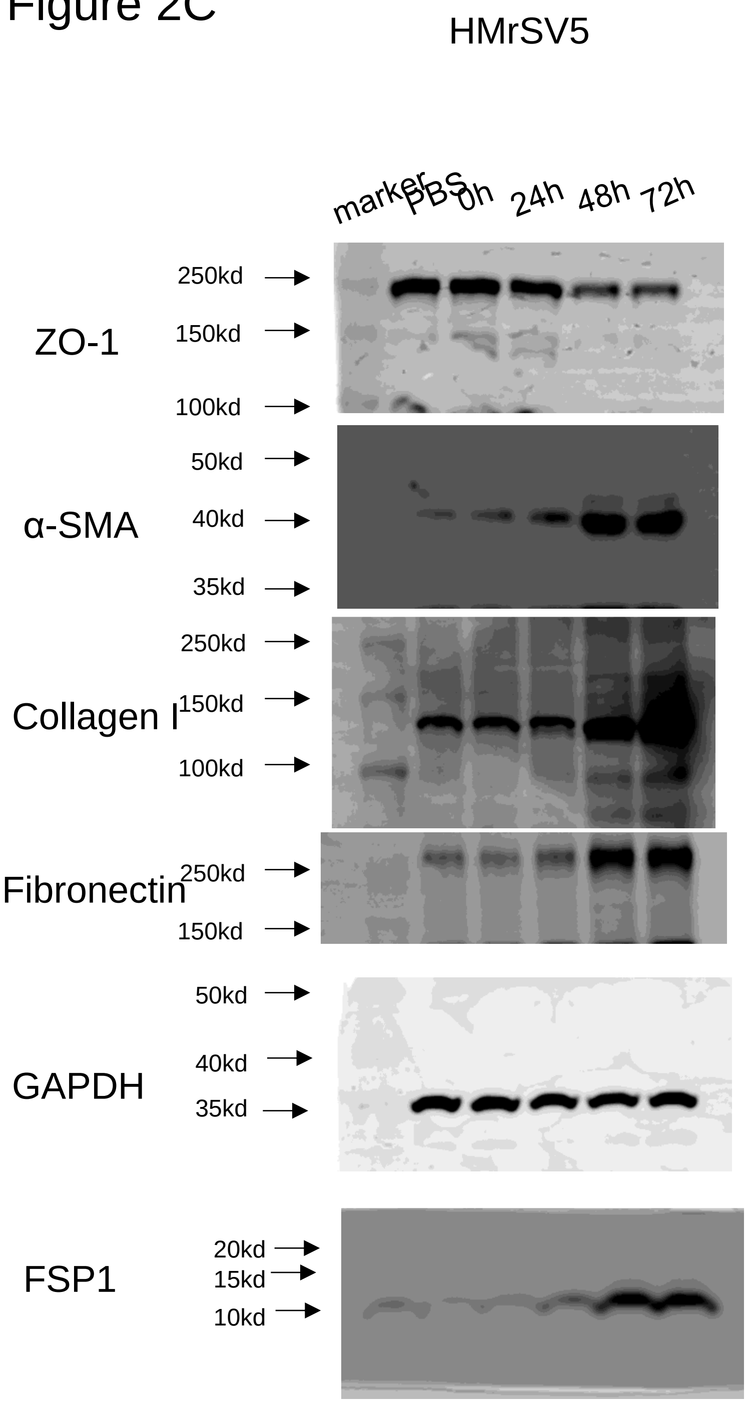
HMrSV5
MET-5A
marker
PBS
0h
24h
48h
72h
ZO-1
α-SMA
Collagen I
Fibronectin
GAPDH
FSP1
250kd
150kd
100kd
50kd
40kd
35kd
20kd
15kd
10kd

Figure 3F

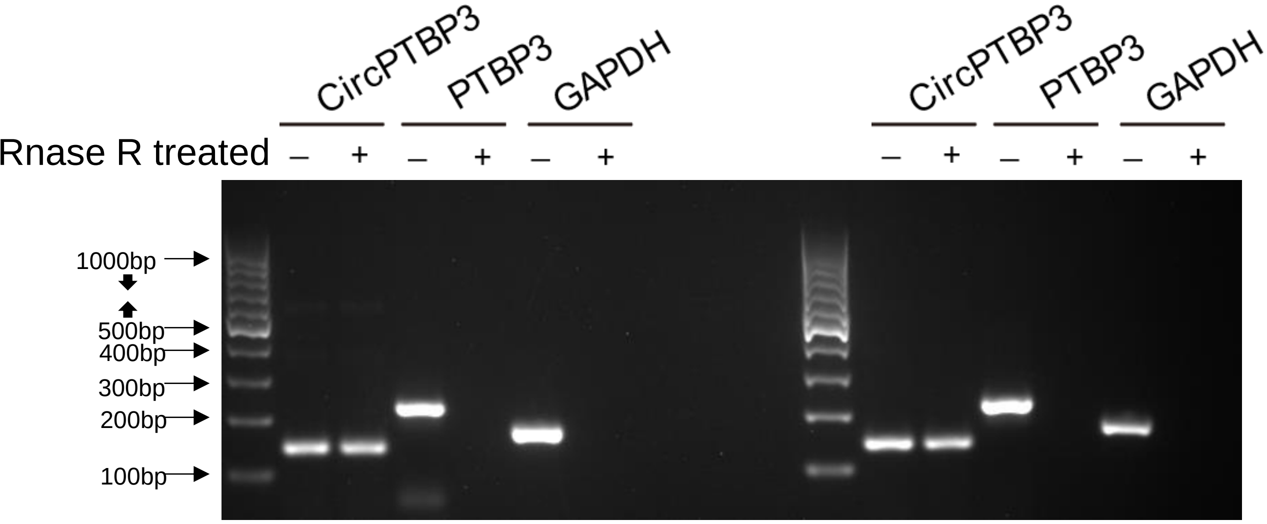
HMrSV5
MET-5A
CircPTBP3
PTBP3
GAPDH
CircPTBP3
PTBP3
GAPDH
Rnase R treated
– + – + – + – + – + – +
1000bp
500bp
400bp
300bp
200bp
100bp

Figure 4C

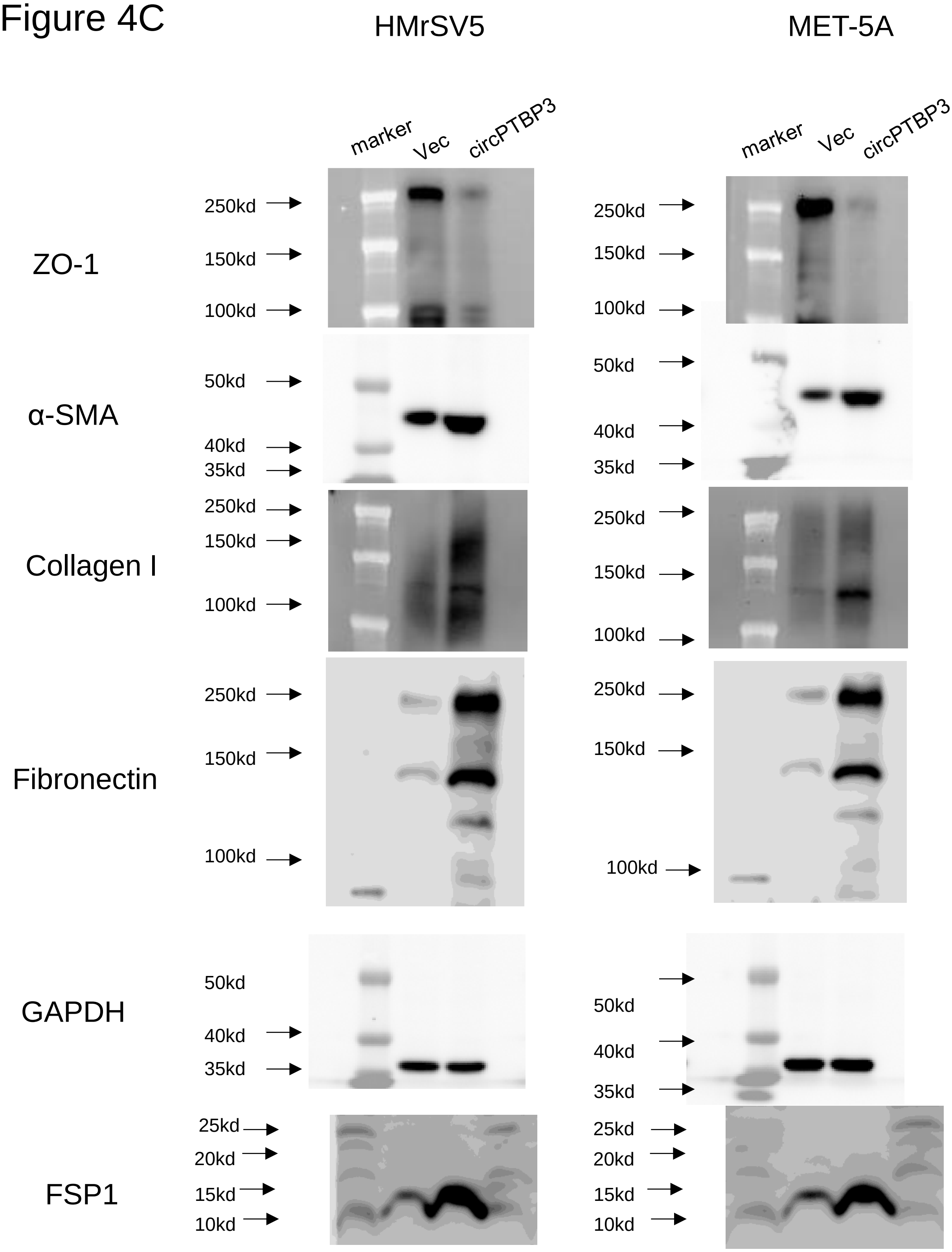
HMrSV5
MET-5A
marker
Vec
circPTBP3
ZO-1
α-SMA
Collagen I
Fibronectin
GAPDH
FSP1
250kd
150kd
100kd
50kd
40kd
35kd
25kd
20kd
15kd
10kd

Figure 5F

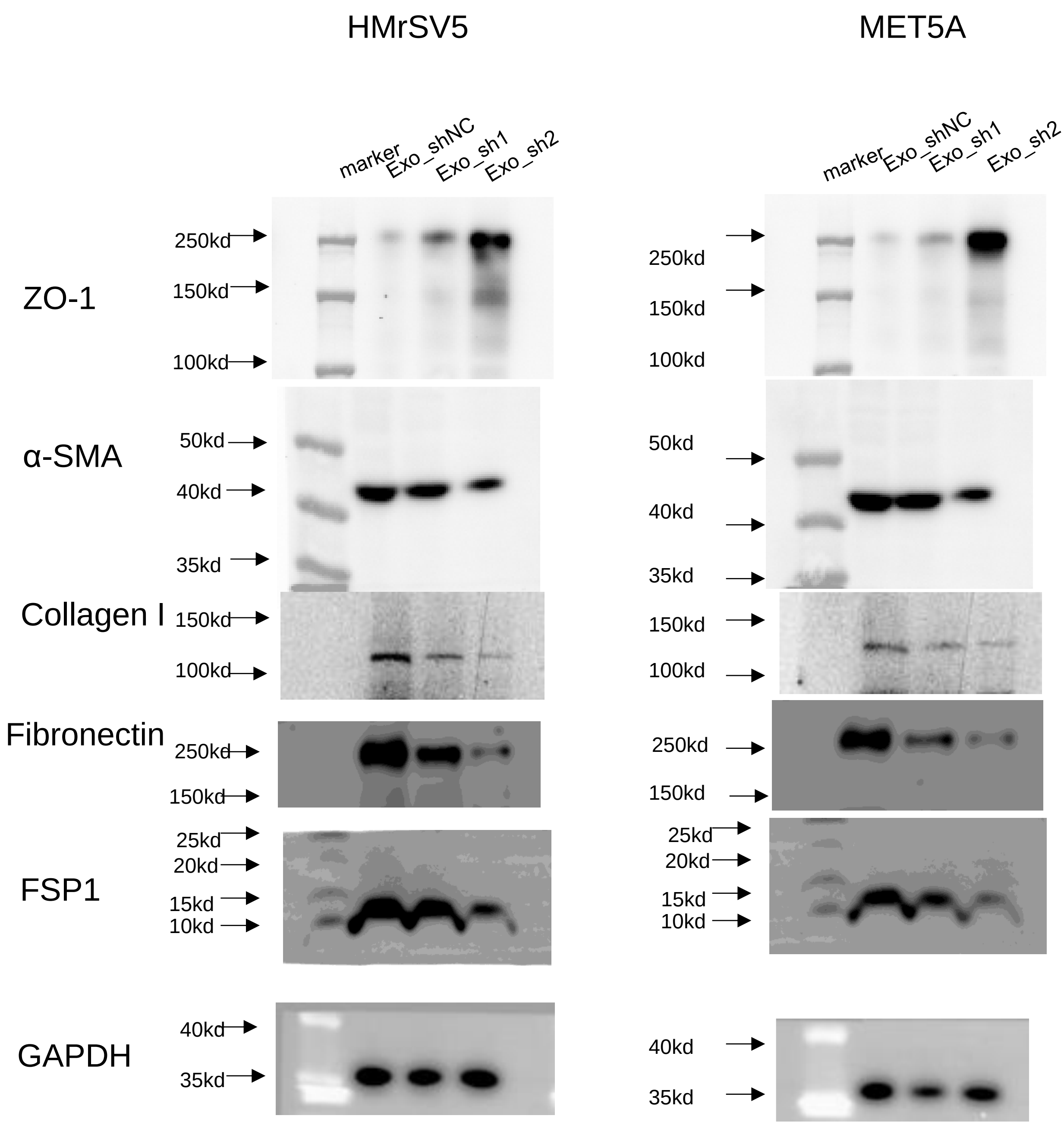
HMrSV5
MET5A
marker
Exo_shNC
Exo_sh1
Exo_sh2
ZO-1
α-SMA
Collagen I
Fibronectin
FSP1
GAPDH
250kd
150kd
100kd
50kd
40kd
35kd
25kd
20kd
15kd
10kd

Figure 6E

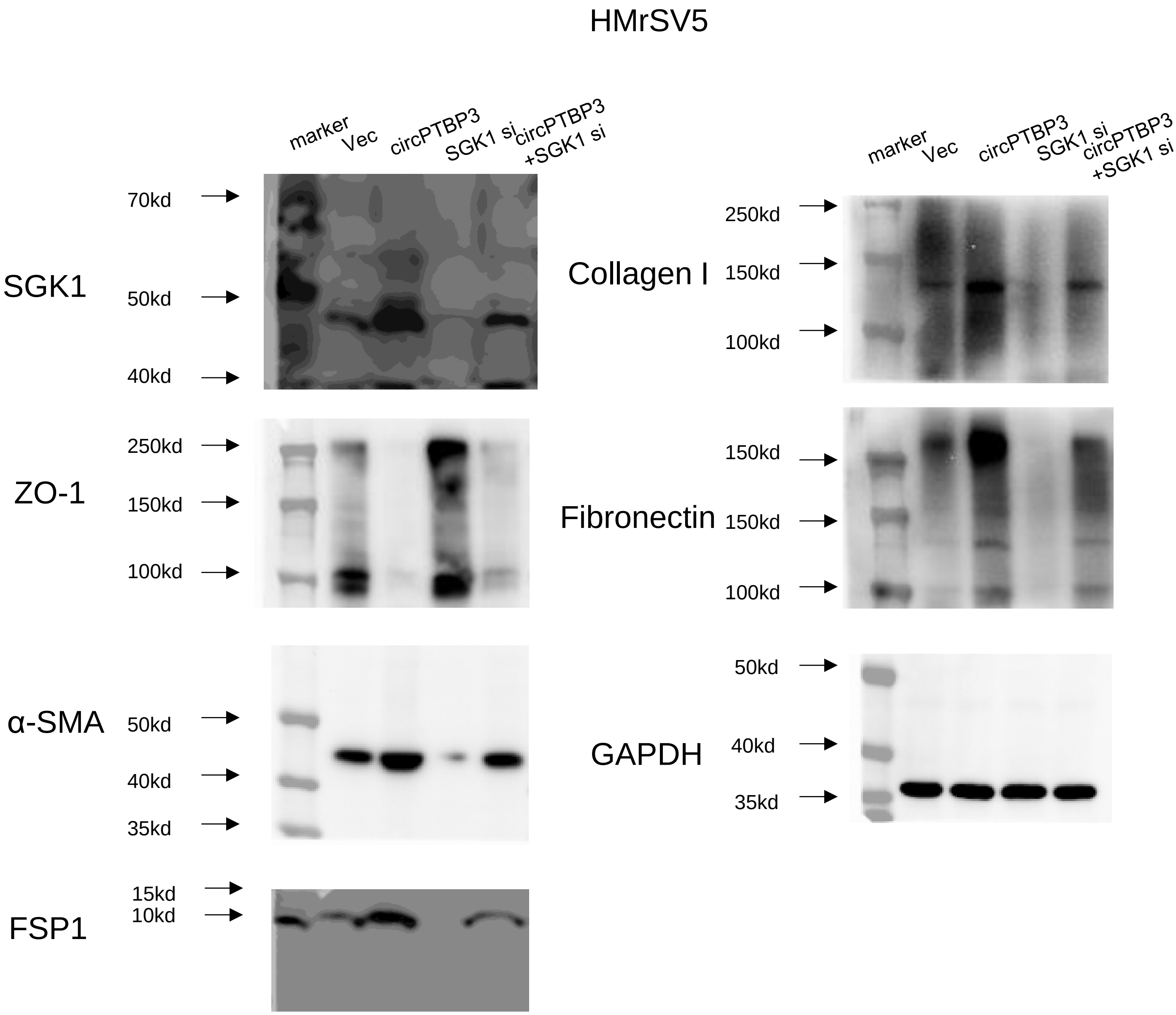

HMrSV5
marker
Vec
circPTBP3
SGK1 si
circPTBP3
+SGK1 si
SGK1
70kd
50kd
40kd
ZO-1
250kd
150kd
100kd
α-SMA
50kd
40kd
35kd
FSP1
15kd
10kd
Collagen I
250kd
150kd
100kd
Fibronectin
150kd
150kd
100kd
GAPDH
50kd
40kd
35kd

Figure 6E

MET5A

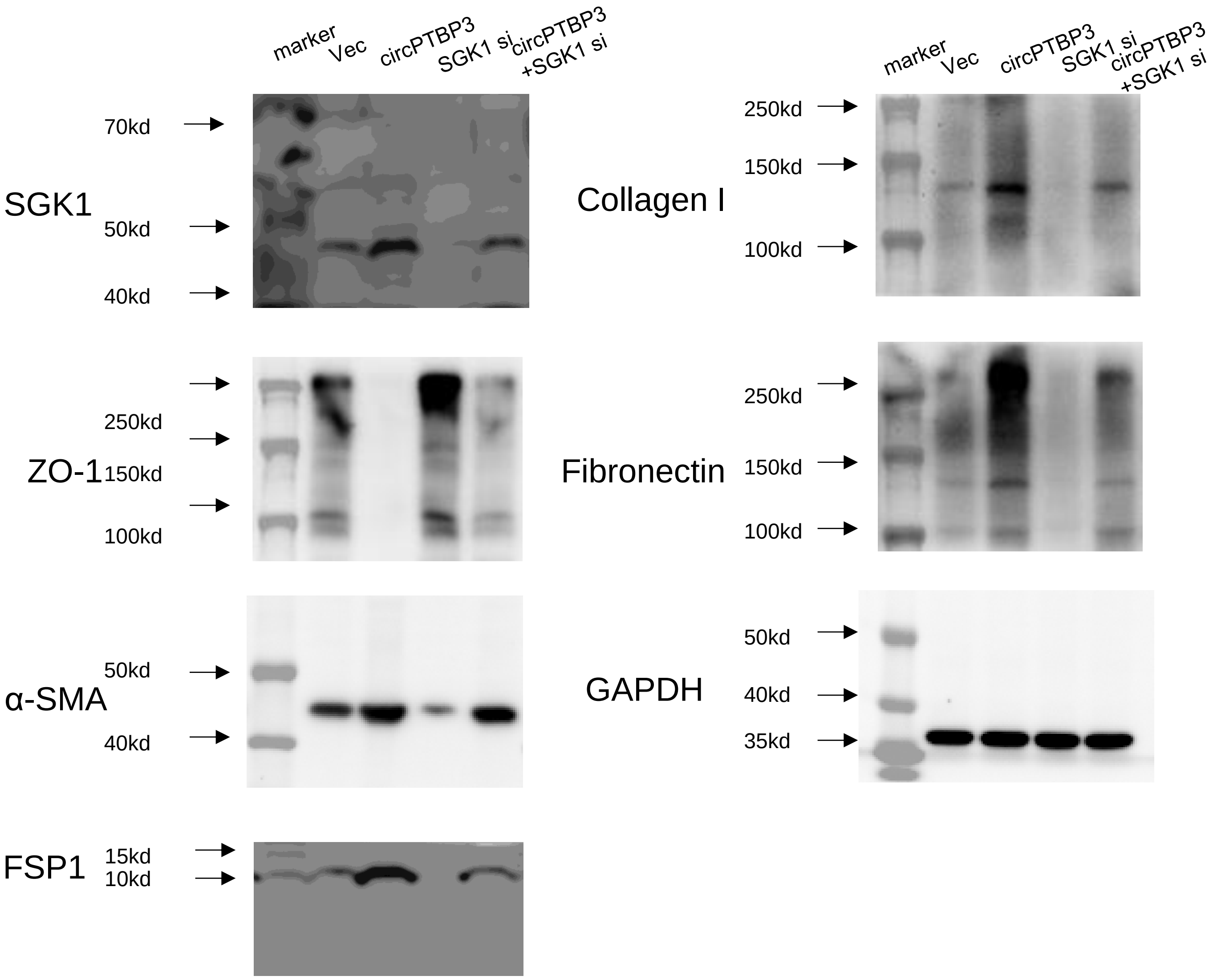

Figure 7C

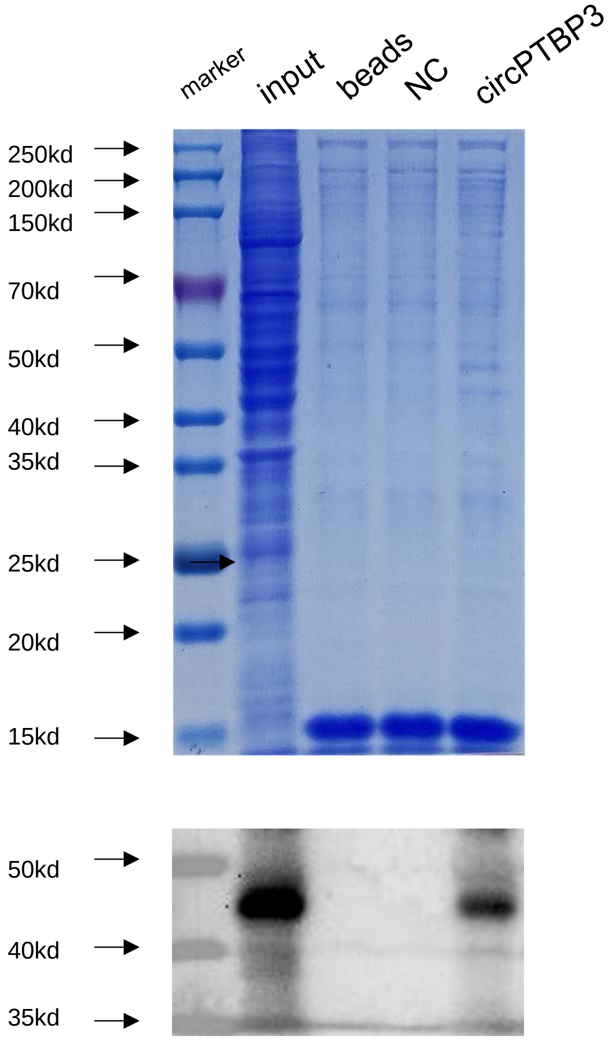

IB:TFAP2B

Figure 7D

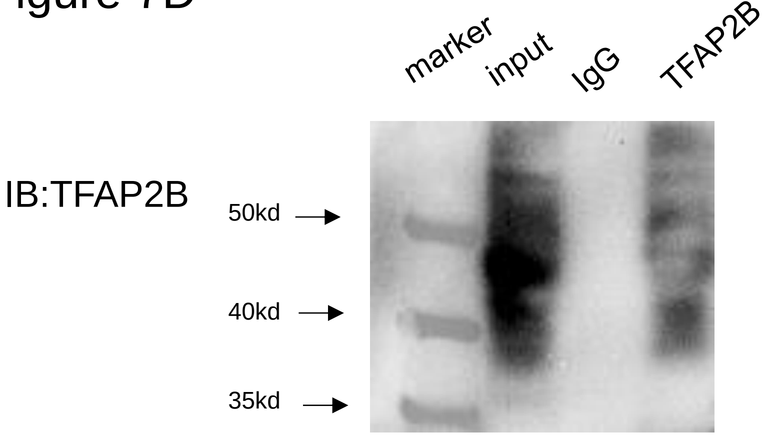

Figure 7E

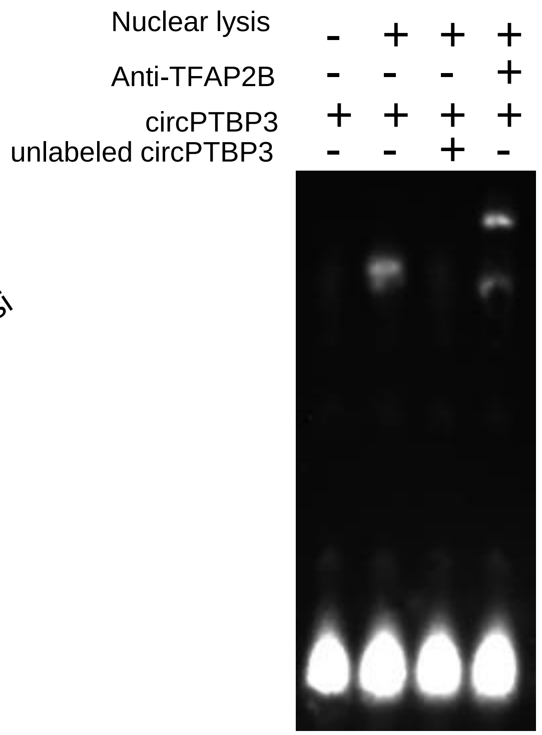

Figure 7K

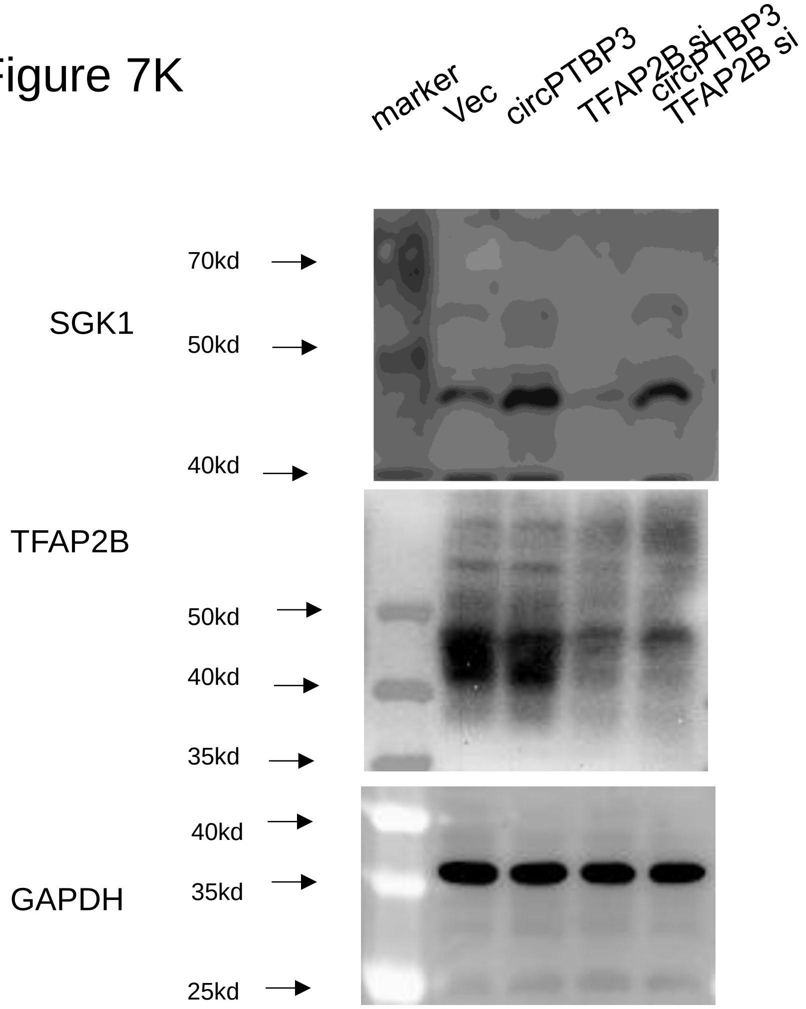

Figure S2C

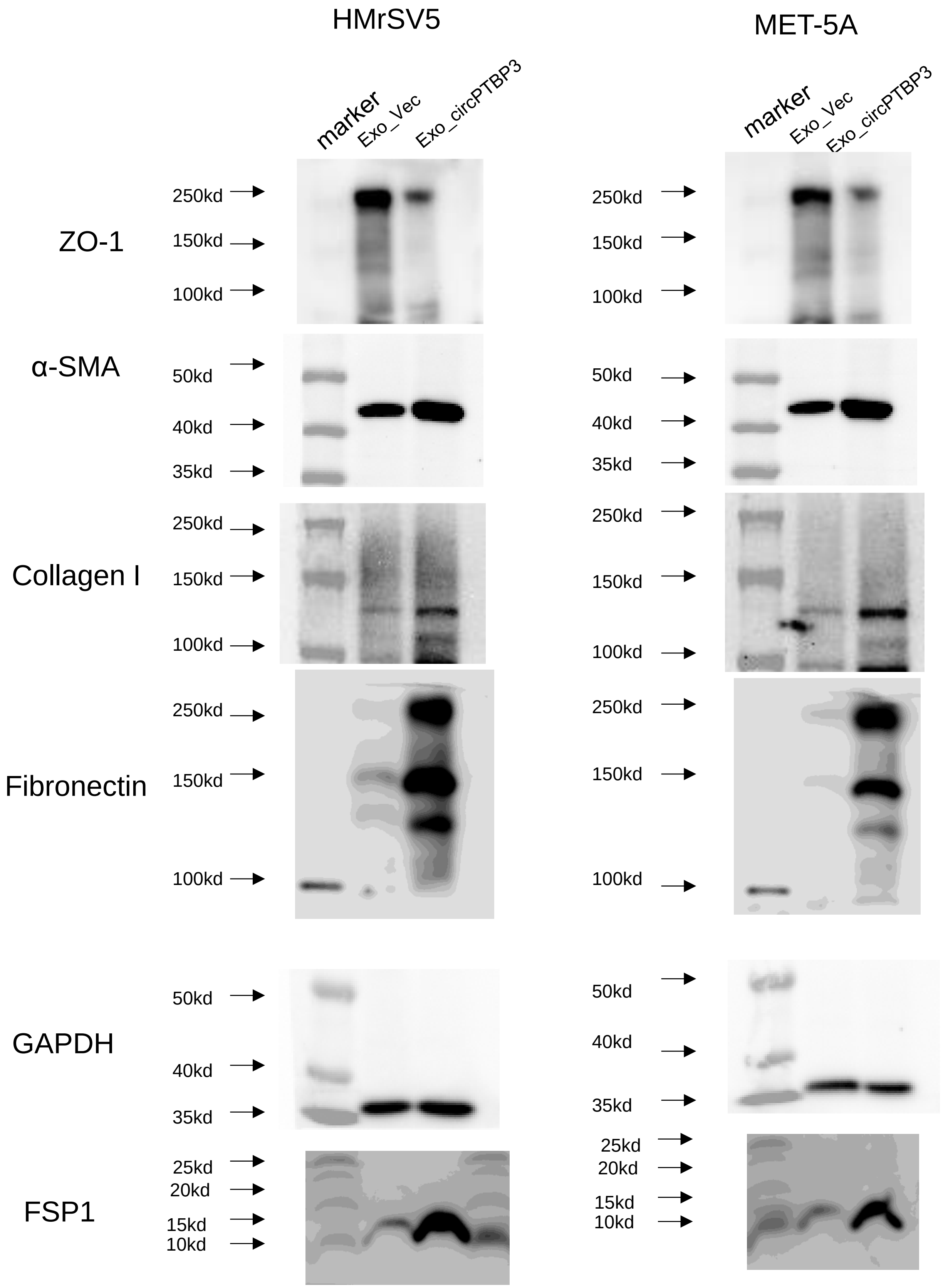
HMrSV5
MET-5A
marker
Exo_Vec
Exo_circPTBP3
ZO-1
α-SMA
Collagen I
Fibronectin
GAPDH
FSP1
250kd
150kd
100kd
50kd
40kd
35kd
25kd
20kd
15kd
10kd
