# Supplementary material for: Tumor exosomal circPTBP3 drives gastric cancer peritoneal metastasis via mesothelial-mesenchymal transition
Source: Cell Death Dis. 2025 Jun 11;16(1):444. doi: 10.1038/s41419-025-07749-z (PMC12159144; doi:10.1038/s41419-025-07749-z)
Supplement: Supplementary file 2 — Supplementary Figures and Tables [file 41419_2025_7749_MOESM2_ESM.pdf]

Supplementary Materials for

Tumor-derived circPTBP3 promotes gastric cancer peritoneal metastasis by inducing mesothelial to mesenchymal transition

This PDF file includes:

Figs. S1 to S8
Tables S1 to S4

Figures and Tables

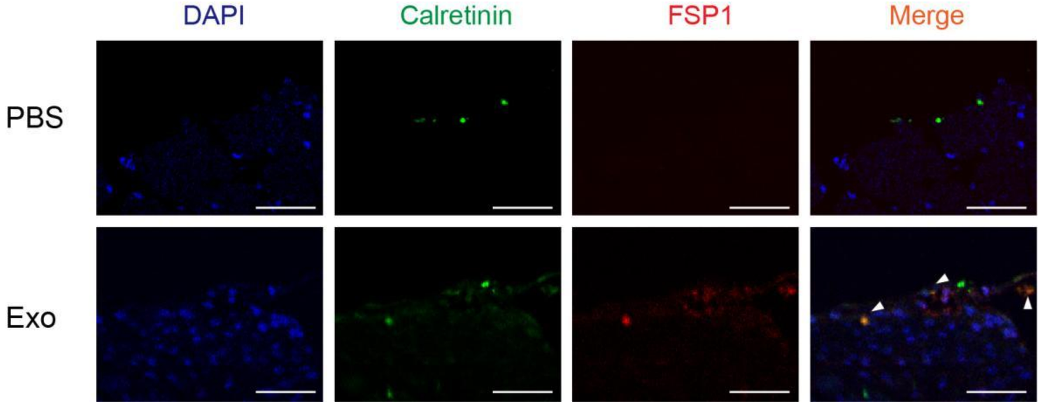

Fig. S1. GC exosomes promoted the infiltration of CAFs in the peritoneum. Immunofluorescence staining of the peritoneum of mice. Blue, DAPI, nucleus; green, calretinin, marker for mesothelial cell; red, FSP1, marker for CAFs. Scale bars, 50 μm.

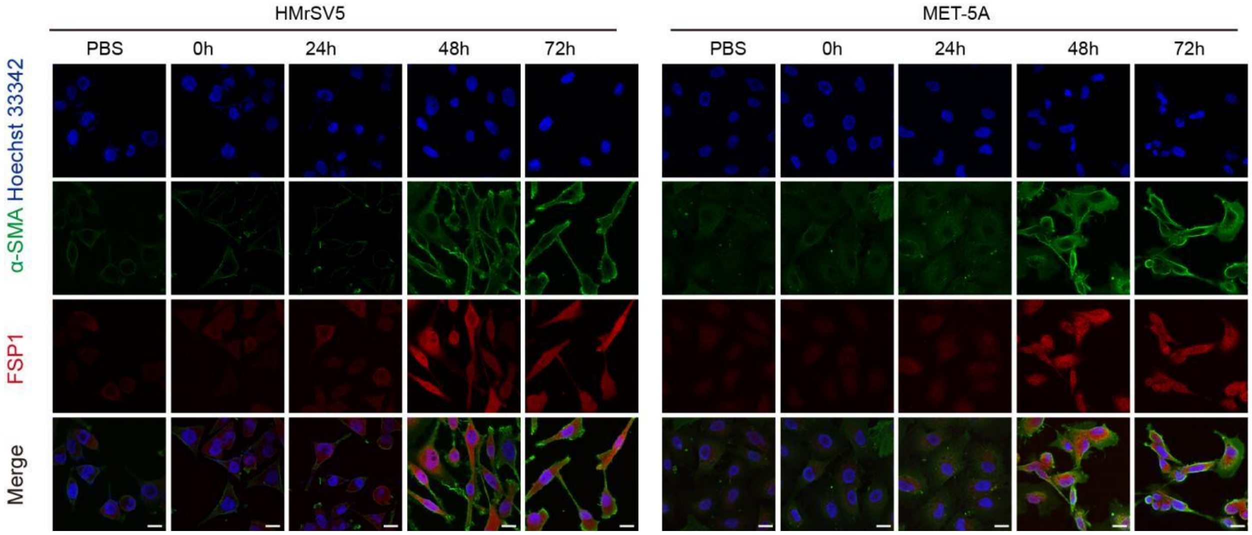

Fig. S2. GC exosomes promoted the transformation of mesothelial cells into CAFs. Immunofluorescence staining of mesothelial cells following co-culture with GC exosomes. Blue, Hoechst33342, nucleus; green, α-SMA; red, FSP1. Scale bars, 20 μm.

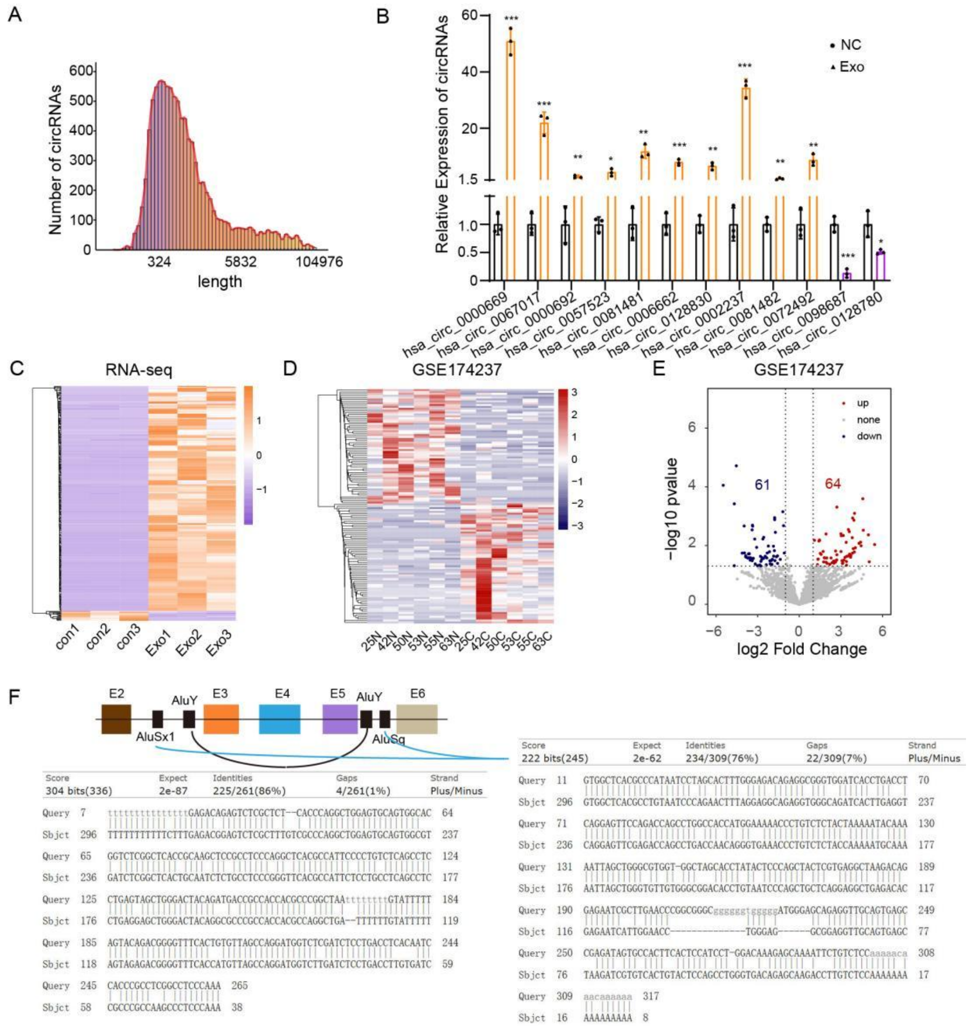

Fig. S3. RNA-seq analysis. (**A**) CircRNA length distribution. (**B**) Checking the quality of RNA-seq with the top 10 up-regulated circRNAs and 2 down-regulated circRNA by PCR analysis. (**C**) Heat map of differential expressed circRNAs in RNA-seq. (**D-E**) Heat map and volcano map of differential expressed circRNAs in GSE174237. (**F**) Representative alignment of reverse complementary sequences of circPTBP's flanking Alu elements. (*, p < 0.05; **, p < 0.01; ***, p < 0.001; ns, no significance)

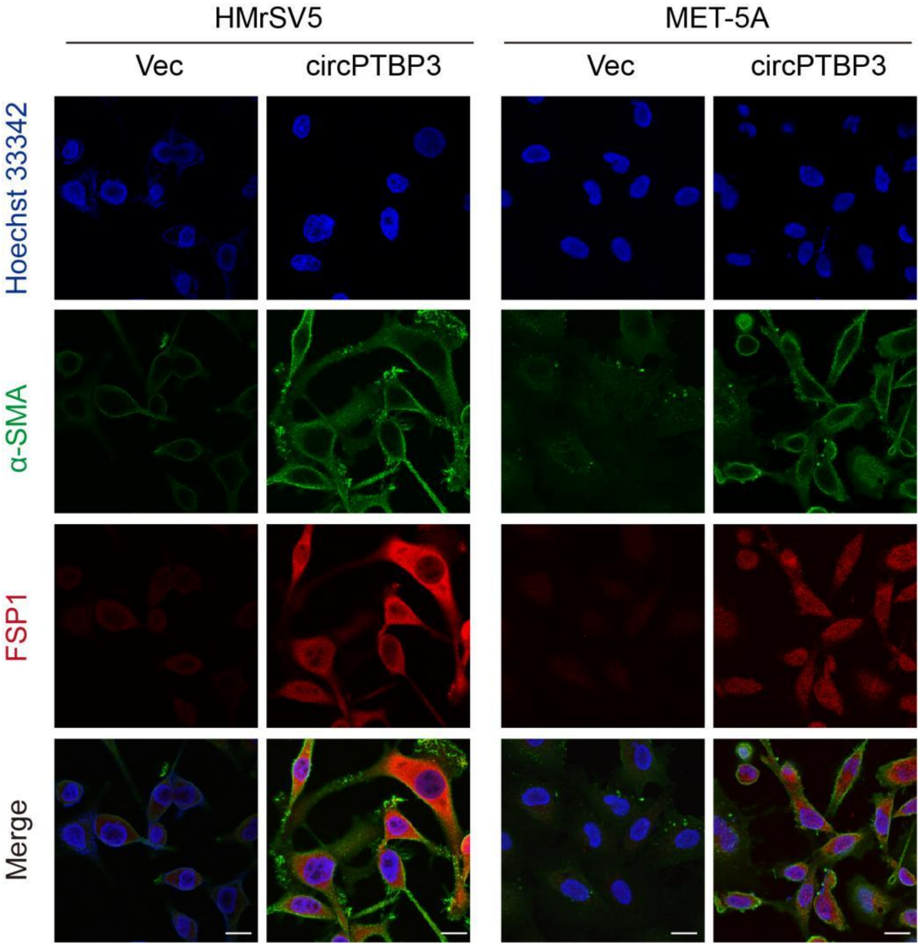

Fig. S4. CircPTBP3 overexpression promoted the transformation of mesothelial cells into CAFs. Immunofluorescence staining of mesothelial cells over expressing circPTBP3. Blue, Hoechst33342, nucleus; green, α-SMA; red, FSP1. Scale bars, 20 μm.

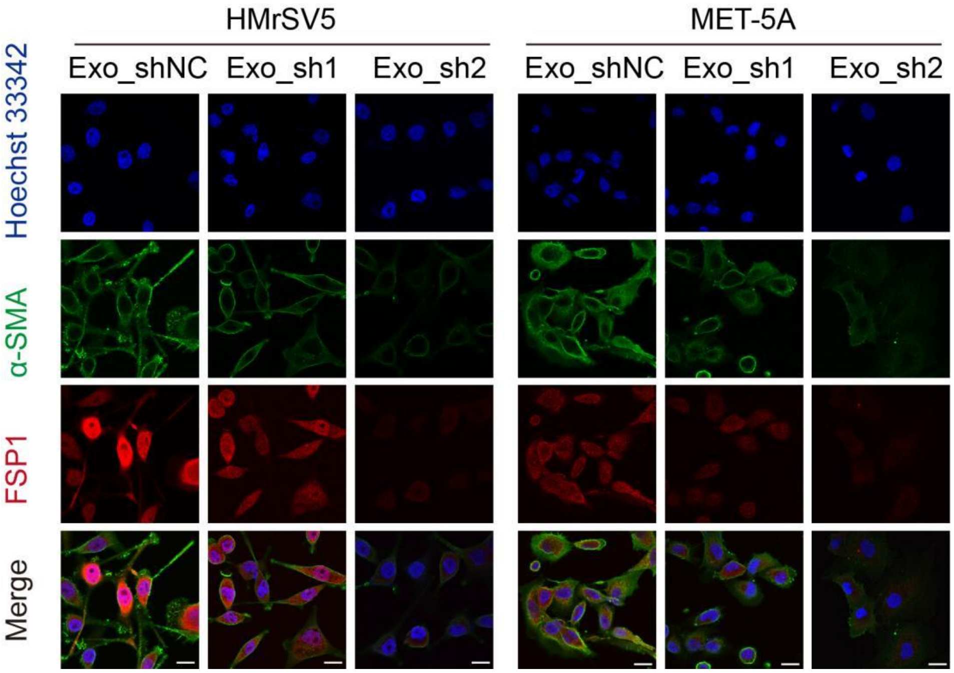

Fig. S5. Exosomes derived from circPTBP3 knockdown MGC803 cells reduced their ability to induce the expression of CAF markers in mesothelial cells. Immunofluorescence staining of mesothelial cells following co-culture with MGC803 exosomes. Blue, Hoechst33342, nucleus; green, α-SMA; red, FSP1. Scale bars, 20 μm.

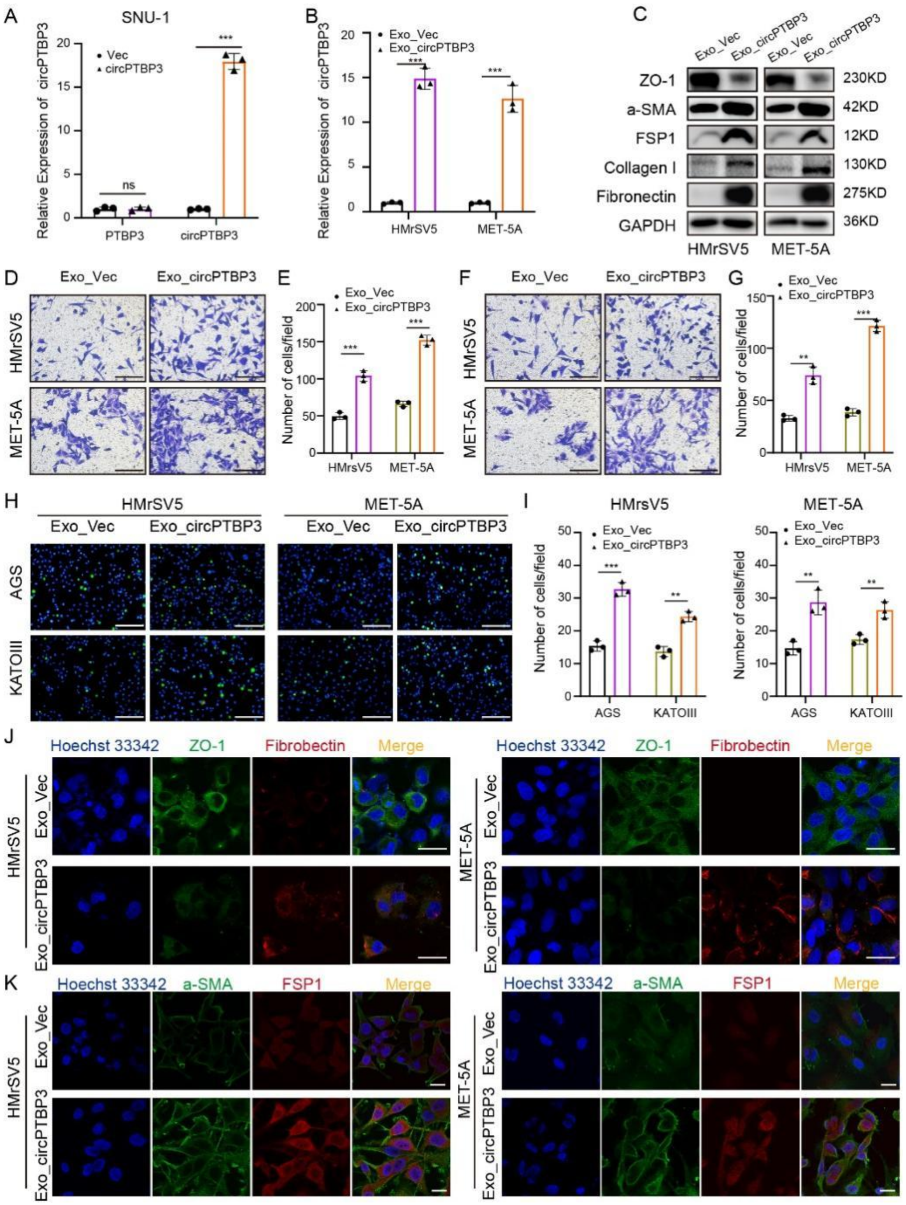

Fig. S6. Exosomes extracted from circPTBP3 over-expression GC cells effectively promoted the MMT of mesothelial cells *in vitro*. (A) Construction lentivirus-mediated circPTBP3 over-expression SNU-1 cells and its validation by PCR analysis. (B) Detection of circPTBP3 expression in mesothelial cells treated by exosomes from SUN-1 cells in each group. (C) Detection of epithelial and mesenchymal proteins (ZO-1, α-SMA, FSP1, Collagen I and Fibronectin) in mesothelial cells by western blot. (D-E) Observation and statistics of mesothelial cells migrating into the bottom surface of the chamber. Scale bars, 100 μm. (F-G) Observation and statistics of mesothelial cells invading into the bottom surface of the chamber. Scale bars, 100 μm. (H-I) Observation and statistics of GC cells adhering to the mesothelial cell surface. Scale bars, 200 μm. (J) Immunofluorescence imaging of epithelial and mesenchymal markers of mesothelial cells. Blue, Hoechst33342, nucleus of mesothelial cells; green, ZO-1; red, fibronectin. Scale bars, 10 μm. (K) Immunofluorescence imaging of CAFs markers. Blue, Hoechst33342, nucleus of mesothelial cells; green, α-SMA; red, FSP1. Scale bars, 20 μm. (*, $p < 0.05$; **, $p < 0.01$; ***, $p < 0.001$; ns, no significance)

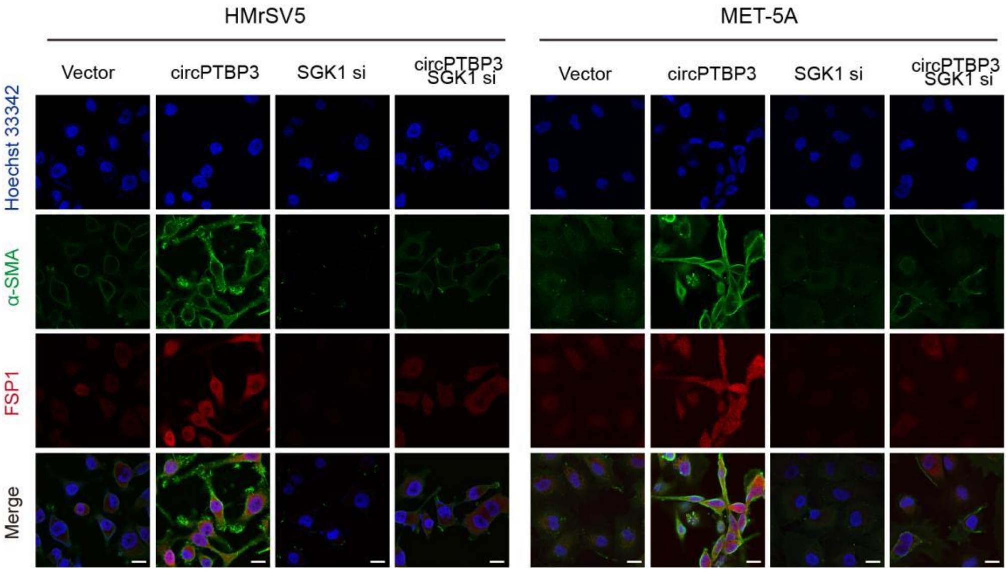

Fig. S7. Knockdown of SGK1 inhibits the transformation of mesothelial cells into CAFs mediated by circPTBP3. Immunofluorescence staining of mesothelial cells. Blue, Hoechst33342, nucleus; green, α-SMA; red, FSP1. Scale bars, 20 μm.

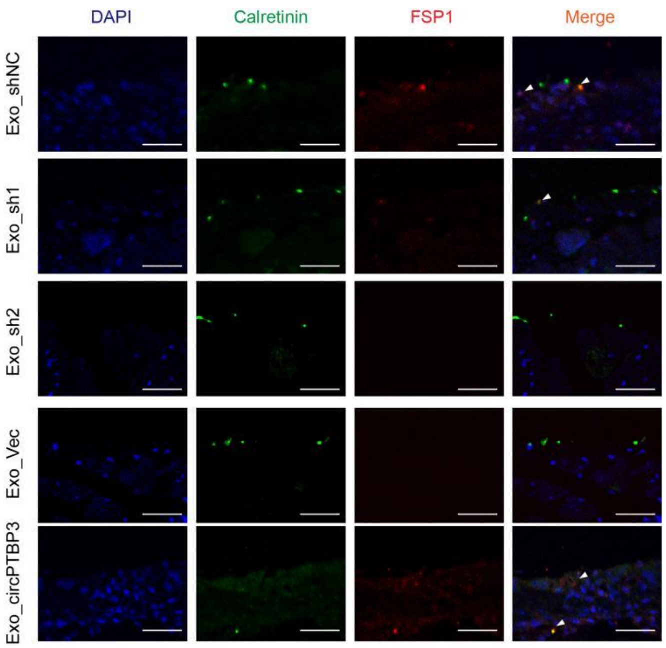

Fig. S8. Exosomal circPTBP3 promoted the infiltration of CAFs in the peritoneum. Immunofluorescence staining of the peritoneum of mice. Blue, DAPI, nucleus; green, calretinin, marker for mesothelial cell; red, FSP1, marker for CAFs. Scale bars, 50 μm.

Table S1. The correlation between clinicopathological parameters and expression of plasma exosomal circPTBP3 in 110 GC patients

Parameters	Cases	circPTBP3 expression		
		Low	High	*p* value
Age (years)				
<60	44	21	23	0.697
⩾60	66	34	32	
Gender				
Female	32	15	17	0.675
Male	78	40	38	
Tumor size (cm)				
<5	48	28	20	0.124
⩾5	62	27	35	
Differentiation				
Moderately-well	45	28	17	0.033*
Poorly	65	27	38	
Depth of invasion				
T1+T2	40	26	14	0.017*
T3+T4	70	29	41	
Lymph node invasion				
No	43	27	16	0.032*
Yes	67	28	39	
Peritoneal metastasis				
No	80	46	34	0.010*
Yes	30	9	21	
TNM stage				
I + II	39	26	13	0.010*
III + IV	71	29	42	

*, p value < 0.05

Table S2. Univariate and multivariate Cox analyses of various potential prognostic factors in 110 GC patients

	**Univariate Cox analysis**		**Multivariate Cox analysis**	
	HR (95%CI)	*p* value	HR (95%CI)	*p* value
Age (⩾60/<60)	1.162(0.720-1.877)	0.539	-	-
Gender(male/female)	0.665(0.401-1.102)	0.113	-	-
Tumor size (⩾5cm/<5cm)	1.531(0.945-2.478)	0.083	-	-
Differentiation (poorly/ moderately-well)	2.337(1.403-3.892)	0.001*	1.880(1.105-3.201)	0.020*
Depth of invasion (T3+T4/T1+T2)	1.769(1.066-2.935)	0.027*	1.292(0.748-2.232)	0.358
Lymph node invasion (Yes/No)	1.524(0.939-2.474)	0.088	-	-
Peritoneal metastasis (Yes/No)	1.905(1.159-3.130)	0.011*	1.341(0.763-2.359)	0.308
TNM Stage (III-IV/I-II)	2.692(1.367-5.300)	0.004*	1.388(0.769-2.504)	0.277
circPTBP3 expression (high/low)	2.412(1.493-3.896)	<0.001*	1.763(1.042-2.982)	0.035*

HR: hazard ratio; CI: confidence interval. *, $p < 0.05$.

Table S3. The analysis of top 30 proteins bound by circPTBP3 via mass spectrometry

**Accession**	**Gene Name**	**MW [kDa]**	**-10lgP**
Q92481	TFAP2B_HUMAN	50.4	201.23
Q9Y383	LUC7L2_HUMAN	46.5	186.33
P26368	U2AF2_HUMAN	53.5	173.91
Q14498	RBM39_HUMAN	59.3	164.87
O76021	RSL1D1_HUMAN	54.9	155.51
P08621	SNRNP70_HUMAN	51.5	152.08
P12268	IMPDH2_HUMAN	55.8	148.06
Q8NFW8	CMAS_HUMAN	48.3	147.92
O60832	DKC1_HUMAN	57.6	143.61
P56182	RRP1_HUMAN	52.8	139.67
Q92466	DDB2_HUMAN	47.8	133.65
A0A0J9YVP6	PUF60_HUMAN	57.4	131.24
Q8TDN6	BRIX1_HUMAN	41.4	127.63
Q9Y6W6	DUSP10_HUMAN	52.6	123.87
P55209	NAP1L1_HUMAN	45.3	120.21
P39023	RPL3_HUMAN	46.1	119.34
Q05519	SRSF11_HUMAN	53.5	117.24
Q8WXA9	SREK1_HUMAN	59.3	114.1
P60709	ACTB_HUMAN	41.7	112.83
F5H5D3	TUBA1C_HUMAN	57.7	109.14
Q9NQ29	LUC7L_HUMAN	43.7	106.81
P68366	TUBA4A_HUMAN	49.9	105.94
A0A2R8Y5V0	SH3BP1_HUMAN	40.3	96.33
Q8NC51	SERBP1_HUMAN	44.9	95.99
Q9UQ80	PA2G4_HUMAN	43.8	90.92
J3KPP4	LUC7L3_HUMAN	58.2	88.73
Q16630	CPSF6_HUMAN	59.2	87.64
H7C2Q8	EBNA1BP2_HUMAN	40.7	82.24
J3KNI5	TRIML2_HUMAN	48.1	80.21
A0A3B3ITJ4	HNRNPL_HUMAN	59.2	79.79

Transcription factors are marked in red.

Table S4. Primers for PCR analysis included in this study

Primer Name	Sequence, 5'-3'
circPTBP3-FP	TCTGAGGAAGCTGCCGTTAC
circPTBP3-RP	CATCAGATCCCCGAGCTTGA
PTBP3-FP	CTTCTGAGGAAGCTGCCGTT
PTBP3-RP	GGAAGGACCTCCAGAAAGGG
GAPDH-FP	TCGGAGTCAACGGATTTGGT
GAPDH-RP	TTCCCGTTCTCAGCCTTGAC
SGK1-FP	CCTTCTCCCGCTCGGTAAG
SGK1-RP	AACATATGCATCACCGCTGC
hsa_circ_0000669-FP	TCATTACCCCAGCTGATGGC
hsa_circ_0000669-RP	CCGCAGAGGGGATGGTGAG
hsa_circ_0067017-FP	GTGGCTGAAGAAGTGGTCCT
hsa_circ_0067017-RP	TTCCGGGTTCTGAGCAGTTT
hsa_circ_0000692-FP	GACAACCGGGGTGCTGTTTA
hsa_circ_0000692-RP	CCTCGGGTCTGCTCGTAGTA
hsa_circ_0057523-FP	TGATCCTCGAAGAGAAGGTGG
hsa_circ_0057523-RP	ACTCATCAATGTGTGAGGTAAAAGA
hsa_circ_0081481-FP	CTACGTGGGGACCCTCTTCT
hsa_circ_0081481-RP	CAGGCCCTTCGTGTATCCTT
hsa_circ_0006662-FP	TGGAAAAGAGCCGAGTGGAC
hsa_circ_0006662-RP	AGTCAGGAATCACAAACTTTCTTCT
hsa_circ_0128830-FP	GAACCTTGCCAGCTATACCC
hsa_circ_0128830-RP	ACATACAGCCACGTAATCCAG
hsa_circ_0002237-FP	ATCTAGACCCACAAGGTCCC
hsa_circ_0002237-RP	CTTCAGATCCCCTCTTGGGAAG
hsa_circ_0081482-FP	CCCTCTACGTCACAGACACG
hsa_circ_0081482-RP	GTGATCCTTGGTGGGCAAGA
hsa_circ_0072492-FP	AGTTAAGGAAGGCAGCTGGA
hsa_circ_0072492-RP	ACGCATAAGGTATGGTGTCTTCT
hsa_circ_0098687-FP	GAATTCGCAAGCTGCGTGA
hsa_circ_0098687-RP	TCACTTGGCATCTGGCCATC
hsa_circ_0128780-FP	TCAGAGCGTGTATGGAGTGG
hsa_circ_0128780-RP	TGGTTACTGCACTTGACGGAG
CCL2-FP	AGCAGCAAGTGTCCCAAAGA
CCL2-RP	TTGGGTTTGCTTGTCCAGGT
EGF-FP	ATGTGTGCAGAGGGATACGC
EGF-RP	CTACAGGGCACGTGCAGTAA
F2-FP	GCATCGTCTCATGGGGTGAA
F2-RP	TTCACGGGATTGGTTCCAGG
GLI1-FP	GGTCCTGGGGGTGCAATAAG

GLI1-RP	GCCCCTCACCTCCCTTCTAT
HES1-FP	TTTCCTCATTCCCAACGGGG
HES1-RP	GGTGGGTTGGGGAGTTTAGG
IL1B-FP	CAGAAGTACCTGAGCTCGCC
IL1B-RP	AGATTCGTAGCTGGATGCCG
MMP1-FP	TGTGGTGTCTCACAGCTTCC
MMP1-RP	CGCTTTTCAACTTGCCTCCC
SNAI1-FP	GTTTACCTTCCAGCAGCCCT
SNAI1-RP	TCCCAGATGAGCATTGGCAG
TNF-FP	CAAGGACAGCAGAGGACCAG
TNF-RP	TCCTTTCCAGGGGAGAGAGG
VEGFA-FP	TGTCTAATGCCCTGGAGCCT
VEGFA-RP	TAACTCAAGCTGCCTCGCC
SGK1_Promoter-100~-300_FP	GACAAGTAGGGGAGGGGAGG
SGK1_Promoter-100~-300_RP	CACACCGTTTTTATTTTATGCTCA
SGK1_Promoter-400~-600_FP	AAAATGGAAAAATAAGCCACATACA
SGK1_Promoter-400~-600_RP	ATTACCAAAAAGTACTACTGAATCC
SGK1_Promoter-700~-900_FP	AAGGCTCCACATCCTGAGGACAC
SGK1_Promoter-700~-900_RP	CCAACATCGGTGTGTGGCTTTC
SGK1_Promoter-1000~-1200_FP	GTTATCATGCCATCATTTAGTTGT
SGK1_Promoter-1000~-1200_RP	AAGTATTTACATGGAAGTCTCTGGT
SGK1_Promoter-1300~-1500_FP	GGGACGAAAAAGAAAGAACTTGAAT
SGK1_Promoter-1300~-1500_RP	TTTGTGGTTCTTCTAAAGAGTTCAT
SGK1_Promoter-1600~-1800_FP	ACCAGTATGCATGCTAGGCCAAT
SGK1_Promoter-1600~-1800_RP	CTTTTTCGTCCCATTGCCACTC
SGK1_Promoter-1900~-2100_FP	CAAGAGGTGGCATGGAGTGA
SGK1_Promoter-1900~-2100_RP	GTGCAGGCGGGTGGTTAATA
SGK1_Promoter-2200~-2400_FP	ACACTGTATATTTCTCTACTAGGCT
SGK1_Promoter-2200~-2400_RP	GGTCCAGTGCATGTAAGTGT
SGK1_Promoter-2500~-2700_FP	CCTACTCATTCTTTAAGGCCATGC
SGK1_Promoter-2500~-2700_RP	TTGGAATGTATCCCCCATGGAT
SGK1_Promoter-2800~-3000_FP	ATTATAAGCAGGAGCCTGTGC
SGK1_Promoter-2800~-3000_RP	GCATTAACTGCACTGTGTTGGT
TFAP2B-FP	AAATCGGTTTGAATTTACCCGC
TFAP2B-RP	CTTCGTGAAATGGCCACACG
U6-FP	CGCTTCGGCAGCACATATAC
U6-RP	AGGGGCCATGCTAATCTTCT
18S-FP	CGGCGACGACCCATTCGAAC
18S-RP	GAATCGAACCCTGATTCCC
